# Microproteins in Metabolic Biology: Emerging Functions and Potential Roles as Nutrient-Linked Biomarkers

**DOI:** 10.3390/ijms262411883

**Published:** 2025-12-09

**Authors:** Seong-Hee Ko, BeLong Cho, Dayeon Shin

**Affiliations:** 1 Department of Food and Nutrition, Inha University, 100 Inha-Ro, Michuhol-Gu, Incheon 22212, Republic of Korea; maria625@inha.ac.kr; 2 Department of Family Medicine, Seoul National University Hospital, 101 Daehak-Ro, Jongno-Gu, Seoul 03080, Republic of Korea; belong@snu.ac.kr

**Keywords:** microproteins, nutrient sensing, mitochondrial regulation, metabolic reprogramming, precision nutrition, obesity, type 2 diabetes, metabolic disorder

## Abstract

Microproteins are small polypeptides translated from short open reading frames (sORFs) that typically encode < 100 amino acids. Advances in ribosome profiling, mass spectrometry, and computational prediction have revealed a growing number of microproteins that play important roles in cellular metabolism, organelle function, and stress adaptation; however, these were considered non-coding or functionally insignificant. At the mitochondrial level, microproteins, such as MTLN (also known as mitoregulin/MOXI) and BRAWNIN, contribute to lipid oxidation, oxidative phosphorylation efficiency, and respiratory chain assembly. Other microproteins at the endoplasmic reticulum–mitochondria interface, including PIGBOS and several muscle-resident regulators of calcium cycling, show diverse biological contexts in which these microproteins act. A subset of microproteins responds to nutrient availability. For example, SMIM26 modulates mitochondrial complex I translation under serine limitation, and non-coding RNA expressed in mesoderm-inducing cells encoded with peptides facilitates glucose uptake during differentiation, indicating that some microproteins can affect metabolic adaptation through localized translational- or organelle-level mechanisms. Rather than functioning as primary nutrient sensors, these microproteins complement classical nutrient-responsive pathways such as AMP-activated protein kinase-, peroxisome proliferator-activated receptor-, and carbohydrate response element binding protein-mediated signaling. As the catalog of microproteins continues to expand, integrating proteogenomics, nutrient biology, and functional studies will be central to defining their physiological relevance; these integrative approaches will also help reveal their potential applications in metabolic health.

## 1. Introduction

Short open reading frames (sORFs) encode microproteins comprising < 100 amino acids. Because of their short length, these peptides have long been overlooked or misclassified as non-coding sequences in conventional genome annotations [1,2]. In genome annotations, numerous microproteins have been incorrectly classified as non-coding transcripts or omitted. However, over the past decade, accumulating evidence has shown that a substantial number of sORFs are actively translated, giving rise to peptides with distinct biological functions [3,4]. Traditionally, because an ORF of 300 base pairs was essential for protein identification, microproteins were often unintentionally excluded or undervalued during detection and annotation [5]. However, recent advances in cutting-edge genome data analysis technologies, ribosome profiling, microfluidic channels, proteomics techniques, and protein quantitative analysis technologies have revealed that microproteins play biologically important roles in fine-tuning cell signaling pathways within cellular metabolic processes, such as calcium signaling and mechanistic target of rapamycin (mTOR) [6], or in oxidative phosphorylation (OXPHOS) [7] and calcium uptake [8], which attracts attention not only in the field of genome coding but also in research on microproteins.

Notably, the cellular biological functions of the discovered microproteins regulate various metabolic pathways, including metabolism related to energy homeostasis [9], fatty acid and glucose metabolism [10], insulin action and mitochondrial metabolism regulation [9], gene expression and transcription regulation [11], protection from cytotoxicity and oxidative stress [12,13], muscle metabolism and calcium transport, and improvement of cardiac muscle function [14]. Thus, microproteins are significantly related to metabolic disorders, obesity, mitochondrial regulation, muscle disease, and heart disease, and are attracting attention as promising targets for future therapeutic agents and biomarker development.

Recent studies have revealed that microproteins act as nutrient-responsive regulators at translational or organelle levels and can instantly link nutrient availability to metabolic activity at the translational or organelle level [15]. This novel mechanism contrasts with traditional nutrient-sensing models centered on signal transduction kinases and transcription factors, such as AMP-activated protein kinase (AMPK), peroxisome proliferator-activated receptor (PPAR), carbohydrate response element binding protein (ChREBP), and hypoxia-inducible factor-1 (HIF-1) [16], which indirectly operate through multistep regulatory pathways controlling post-translational modification and chromatin remodeling, resulting in delayed and redundant signaling responses [17].

In classical models, AMPK responds to increased AMP/ATP ratios under energy stress by promoting glucose uptake, fatty acid oxidation, and autophagy [18]. PPAR acts as a lipid sensor that regulates fatty acid metabolism [19]. ChREBP senses carbohydrates to activate the transcription of genes involved in glycolysis and lipogenesis [20], whereas HIF-1 functions in hypoxia and nutrient limitation by inducing glycolytic genes and angiogenesis [21]. Although these factors form a fundamental framework for understanding metabolic homeostasis and disease, the signaling- and transcription-centered paradigm inherently involves indirect and delayed responses.

**Figure 1:**
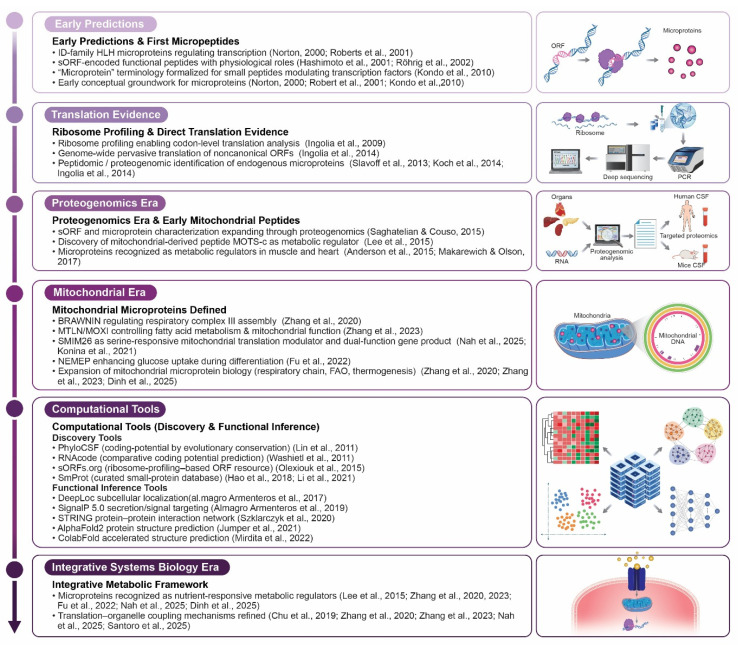
Historical timeline of microprotein discovery and technological advances [22,26,28,29,31,32,33,36,37,38,40,41,42,43,45,46,47,48,49,50,51,52,53,54,55,56,57,58,59,60,61,62]. All schematic figures were generated with Adobe Illustrator 2025.

In contrast, microproteins act upstream of gene expression and modulate metabolic functions immediately through translational control or interactions within cellular organelles. This means that without waiting for complex transcription (the process by which genes are copied into RNA), they can immediately alter the metabolic functions of the cell by directly regulating them at the protein translation stage or by acting directly inside organelles such as the mitochondria. For example, the serine-responsive mitochondrial microprotein, SMIM26, regulates mitochondrial ribosomal translation in response to amino acid availability, thereby controlling complex I activity [22]. Similarly, the nodal-enhanced microprotein, non-coding RNA expressed in mesoderm-inducing cells encoded with peptide (NEMEP), promotes glucose uptake during embryonic differentiation through direct interaction with glucose transporter proteins (GLUTs) [23]. These findings highlight the translational layer of nutrient-responsive regulation that complements transcriptional mechanisms and links fluctuations in nutrient availability with rapid adjustments in energy production and cellular metabolism. In addition to these functional characteristics, microproteins exhibit distinctive biochemical properties that are essential for interpreting their metabolic roles. Microproteins generally constitute a highly dynamic, low-abundance layer of the proteome, reflecting their short half-lives, rapid turnover, and context-dependent expression. Quantitative proteomic analyses estimate that most microproteins account for less than 0.1% of total cellular protein mass, consistent with their small molecular size and susceptibility to proteasome-dependent degradation [24,25]. Because many microproteins contain intrinsically disordered regions rather than stable tertiary structures, they often display fast on/off expression dynamics and transient accumulation in response to nutrient fluctuations or stress cues. These biochemical features provide important context for the temporal framework illustrated in Figure 1, in which microproteins operate as rapid, translation-proximal modulators of metabolic activity, clearly distinct from the slower transcription-driven remodeling mediated by canonical nutrient-sensing pathways.

Despite their small size and frequent intrinsic disorder, many microproteins adopt functional microdomains such as amphipathic α-helices, β-hairpin-like motifs, and short linear motifs that enable fast, reversible protein–protein or membrane interactions [26,27]. Structural predictions using AlphaFold2 and ColabFold indicated that several mitochondrial microproteins (e.g., MTLN, BRAWNIN, and SMIM26) undergo disorder-to-order transitions upon binding or membrane association, suggesting that inducible structural plasticity supports their regulatory roles [28,29]. This explains why microproteins function effectively as rapid metabolic modulators despite lacking classical globular folds [22,30].

Over the past two decades, the conceptual landscape of microproteins has undergone a profound transformation. Early foundational studies, including the discovery of the mitochondria-derived peptide, humanin (HN), in 2001 as a cytoprotective micropeptide, and the identification of ENOD40-derived regulatory peptides in 2002, first demonstrated that sORFs encode functional peptides with physiological roles [31,32]. In 2010, the term microprotein was later formalized to describe small peptides capable of modulating larger protein complexes, marking a conceptual expansion of peptide-based regulation [33]. In 2011, a major technological breakthrough occurred with the advent of ribosome profiling (Ribo-seq), which provided codon-resolved evidence for the widespread translation of non-canonical sORFs [34]. Subsequently, the first proteogenomic studies between 2013 and 2014 validated dozens of endogenous human microproteins using mass spectrometry combined with ribosome profiling, thereby establishing microproteins as a distinct and functionally relevant layer of the proteome [35,36].

More recently, an expanding suite of computational discovery and functional inference tools, including PhyloCSF [37], sORFs.org [38], DeepRibo [39], SmProt [40], AlphaFold [28], DeepLoc [41], TargetP [42], STRING [43], and BioGRID [44], enabled a large-scale prediction of microprotein-coding ORFs and systematic inference of subcellular localization, structural features, and interaction networks. These advances collectively form a historical and methodological framework that underpins the current understanding of microprotein biology and justifies the integrated perspective presented in this review (Figure 1).

The conceptual and technological evolution of microprotein research from early predictions through the integrative systems biology era. The timeline illustrates key milestones including (1) early predictions with ID-family HLH microproteins and sORF-encoded functional peptides (2000–2010); (2) translation evidence through ribosome profiling and proteogenomic identification (2009–2014); (3) a proteogenomics era with expanding sORF characterization and mitochondrial peptide discovery (2015); (4) mitochondrial era defining microproteins like BRAWNIN, MTLN/MOXI, SMIM26, and NEMEP (2020–2025); (5) computational tools for discovery and functional inference; and (6) integrative systems biology era recognizing microproteins as nutrient-responsive metabolic regulators with translation-organelle coupling mechanisms.

This study introduced microproteins as direct translational nutrient sensors, compared their functions with established regulators, such as AMPK and PPAR, and evaluated their effects on energy metabolism, metabolic diseases, and precision nutrition. Incorporating microproteins into a broader nutrient-sensing framework promotes a conceptual transition from signal transduction to translation-centered metabolic regulation models.

## 2. Discovery of Nutrient-Responsive Microproteins

### 2.1. Overview of sORF-Encoded Microproteins in Nutrient Sensing

Microproteins encoded by sORFs have attracted attention as direct mediators of nutrient sensing and metabolic adaptation. Classic metabolic regulators, including AMPK, PPAR, and ChREBP, whose mechanisms have been elucidated and reported since the 1990s, regulate metabolism through transcription and signaling cascades. AMPK, first reported in 1988, is recognized as a metabolic master switch that monitors the AMP/ATP ratio to detect cellular energy status, and subsequently activates downstream kinases and transcriptional signals [63,64]. Classical nutrient-sensing pathways, such as AMPK, PPARs, and ChREBP, orchestrate metabolic adaptation primarily through signaling cascades and transcriptional remodeling [18,19,20]. In contrast, recently characterized microproteins function at a more proximal level, either through translational regulation or by directly modulating organelle-resident metabolic machinery [22,45]. Rather than replacing canonical pathways, microproteins operate in parallel as fast-acting effectors that fine-tune metabolic responses to nutrient fluctuations [15,22,45]. This complementary relationship provides the conceptual bridge linking classical nutrient sensors to emerging microprotein-mediated regulatory mechanisms [15,45]. PPAR gamma (PPARγ) has been recognized as a principal regulatory factor of adipocyte differentiation and metabolic homeostasis [65]. In the early 2000s, the glucose-responsive transcription factor, ChREBP, was identified, and its involvement in the transcriptional control of genes associated with glycolysis and adipogenesis was subsequently characterized [66]. All of the above classical nutrient sensors function through a cascade of transcriptional and signaling pathways. In addition to ENOD40-derived peptides, another early example that shaped the microprotein concept is the inhibitor of DNA binding (ID) family. ID1–ID4 encode small helix–loop–helix (HLH) regulatory proteins (~13–18 kDa) that act as dominant-negative regulators of basic HLH (bHLH) transcription factors [46,67]. Although historically categorized as transcriptional regulators rather than classical microproteins, small HLHs have been recognized as one of the earliest classes of small regulatory proteins and have helped establish a conceptual basis for defining microproteins as modulators of larger protein complexes [68].

In contrast, microproteins were first reported in 2002 when the plant *Enod40* gene, initially annotated as a long non-coding RNA (lncRNA), was shown to encode two peptides that are 12 and 24 amino acids in length [69]. In 2010, the designation “microprotein” was introduced to describe small peptides capable of modulating the function of larger protein complexes. A well-characterized example is the ID protein, a 16 kDa molecule containing an HLH motif, which is classified as a microprotein and has been demonstrated to inhibit the DNA-binding activity of basic HLH (bHLH) transcription factors by forming non-functional heterodimers, thereby repressing downstream gene expression [15,47]. The ID family, comprising ID1–ID4, comprises evolutionarily conserved microproteins that regulate cell proliferation and differentiation across multiple tissues through negative modulation of bHLH-mediated transcriptional programs [47,70].

What distinguishes many microproteins from classical nutrient sensors is that they often exert rapid, localized actions at the translational or organelle level, although some may also influence transcription indirectly through downstream metabolic or signaling pathways [22,23,45,71]. For example, the serine-responsive mitochondrial microprotein, SMIM26, directly regulates the translation of mitochondrial complex I subunits, linking amino acid availability to OXPHOS without the involvement of transcriptional intermediates [22,72]. Similarly, NEMEP, a microprotein induced during mesendoderm differentiation, increases glucose uptake by physically interacting with GLUT1 and GLUT3, independent of upstream signaling inputs, illustrating how microproteins can modulate nutrient flow to support developmental energy demands [5,23].

Beyond amino acid and glucose sensing, mitochondrial microproteins, such as BRAWNIN and MTLN, integrate nutrient stress with respiratory chain assembly and fatty acid β-oxidation, ensuring cellular energy homeostasis under fluctuating dietary conditions [48,49]; this suggests that in addition to detecting basic nutrients, such as amino acids and glucose, BRAWNIN and MTLN simultaneously regulate the process in which the respiratory chain and fatty acids are utilized as energy sources and mitochondrial stability during energy production, thereby preventing the collapse of the energy-generating system under stressful conditions such as nutrient deprivation or excess. In brown adipose tissue (BAT), microprotein for thermogenesis 1 (MICT1) functions as a nutrient- and diet-responsive thermogenic regulator, thereby amplifying β-adrenergic signaling and synergizing with dietary compounds like caffeine and catechins to promote energy expenditure [50]. Moreover, endocrine-like microproteins, such as neuronatin (Nnat), and the lncRNA-derived β-cell and neuronal lineage regulator (BNLN) couple glucose availability to insulin secretion, highlighting their roles as β-cell nutrient sensors in metabolic control [73].

Recent studies have identified multiple sORF-encoded microproteins that contribute to metabolic adaptations via diverse mechanisms. SMIM26, NEMEP, MTLN, and BRAWNIN represent illustrative examples, acting through serine-responsive mitochondrial translation, glucose-uptake regulation, fatty-acid oxidation, and respiratory-chain assembly, respectively [5,22,23,45,48,49,72]. Other nutrient-responsive microproteins have also been described. For example, MICT1 [50], which integrates dietary and thermogenic cues to enhance β-adrenergic-driven energy expenditure, and BNLN [51], which couples glucose availability to calcium handling and insulin secretion in pancreatic β-cells, both clearly represent nutrient-linked regulation. Another example is Nnat [74], a microprotein whose expression and function are tightly modulated by glucose levels, and it plays a critical role in β-cell endoplasmic reticulum (ER) calcium regulation and insulin release. Importantly, these proteins are part of a broader and rapidly expanding group of microproteins implicated in organelle function, nutrient utilization, and cellular stress responses, suggesting that nutrient-responsive microproteins constitute a heterogeneous class rather than a small set of isolated cases [7,8,9,10,11,12,13,14]. They include regulators of mitochondrial homeostasis, oxidative stress responses, calcium cycling, and other metabolic processes; however, not all of them exhibit nutrient-specific regulation. Collectively, these findings indicate that nutrient-responsive microproteins represent a heterogeneous yet expanding functional class within the microprotein landscape.

Accumulating evidence reveals that microproteins can be broadly grouped into several functional classes:

(1) Mitochondrial metabolic regulators, including MTLN (Mitoregulin/MOXI) and BRAWNIN, which coordinate β-oxidation, OXPHOS, and respiratory chain assembly [24,25];
(2) Calcium- and ion-handling modulators in excitable tissues (e.g., MLN, PLN, SLN, DWORF), which fine-tune SERCA activity and Ca^2+^ cycling [50,75];
(3) Nutrient-responsive translational regulators, such as SMIM26 and NEMEP, which couple amino acid or glucose availability to mitochondrial translation or substrate uptake [76,77];
(4) Stress- and hormone-responsive peptides, such as the mitochondria-derived peptide MOTS-c [52].

Large-scale proteogenomic maps suggest that approximately 35–40% of validated microproteins localize to mitochondria, with the remainder distributed across the ER (15–20%), cytosol (20%), nucleus (10–15%), and inter-organelle contact sites (5–10%) [24,25,77]. These distributions highlight the strong enrichment of microproteins in organelles that directly coordinate metabolic flux and translational activity.

Most microproteins discussed in this review, including SMIM26, NEMEP, MTLN, MOXI and BRAWNIN, are encoded by nuclear genes and synthesized on cytosolic ribosomes. Following translation, they are delivered to mitochondria through either N-terminal targeting sequences or internal targeting motifs that direct their import. In contrast, a minority of microproteins, such as MOTS-c, originate from the mitochondrial genome itself. These differences in genomic origin and import pathways contribute to the diverse subcellular localization and functional specialization of microproteins [25]. In contrast, MOTS-c is encoded by the mitochondrial *12S rRNA* gene and can translocate from mitochondria to the cytosol and nucleus during metabolic stress [75]. These genomic and mechanistic differences highlight the dual origins of microproteins and the diverse import routes that determine their subcellular localization and functional specialization.

Microproteins interact with classical nutrient-sensing pathways through several mechanistic nodes that regulate metabolic flux, translation capacity, and intracellular signaling thresholds [30,63]. Amino acid-responsive microproteins, such as SMIM26, modulate mitochondrial translation of NADH dehydrogenase 5 (ND5) and reshape electron transport activity, thereby influencing ATP/AMP ratios, serine-one-carbon flux, and ultimately, the activation thresholds of AMPK and mammalian target of rapamycin complex 1 (mTORC1) in response to nutrient limitation [22]. In contrast, the glucose-responsive membrane microprotein, NEMEP, facilitates GLUT1/3 trafficking and enhances glucose uptake, positioning it to act in parallel with insulin/AKT signaling, while also modulating the magnitude of downstream glycolytic and biosynthetic responses during developmental transitions [23]. Mitochondrial microproteins, such as MTLN and BRAWNIN, exert their effects through respiratory chain remodeling, affecting complexes I and III assembly, respectively. By altering NAD^+^/NADH ratios, mitochondrial membrane potential, and tricarboxylic acid (TCA)-derived metabolite availability, these microproteins indirectly tune AMPK activation dynamics, mTORC1 sensitivity to mitochondrial stress, and the balance between anabolic and catabolic metabolism [17,30]. Together, these regulatory modes indicate that microproteins operate as translation-proximal, organelle-embedded effectors that refine the amplitude, timing, and metabolic outputs of canonical nutrient-sensing systems rather than functioning as independent or redundant regulators [26].

### 2.2. SMIM26 and Serine Availability of Mitochondrial Translation

SMIM26 is a mitochondrial microprotein translated from the 5′-upstream ORF (5′-uORF) of the *LINC00493* non-coding RNA, and is highly responsive to intracellular serine levels [22]. When serine availability is restricted, cell survival depends on SMIM26 expression. Under these conditions, SMIM26 associates with the mitochondrial serine transporters, sideroflexin 1 and 2 (SFXN1/2), and with the mitoribosome, forming a functional complex that facilitates translation of the mitochondrial complex I subunit ND5 [22]. Loss of SMIM26 expression leads to a decline in mitochondrial serine uptake, accompanied by reduced levels of folate pathway intermediates, and disruption of key tRNA modifications, including τm^5^U and τm^5^s^2^U. Consequently, translation of the ND5 subunit and activity of respiratory complex I are compromised, resulting in a marked reduction in OXPHOS capacity [22]. This results in decreased oxygen consumption, decreased ATP production, and generally, a deficiency in complex I, which severely impairs cellular energy production. Notably, SMIM26 deletion is embryogenic in mouse models, and its tumor growth-inhibitory effects have been reported in xenograft models of folate-dependent acute myeloid leukemia [72].

Notably, these reports highlight the nutritional sensing function of SMIM26 microproteins and draw new attention. SMIM26 functions as a nutrient-responsive regulator highly sensitive to changes in intracellular serine levels. Under serine deprivation conditions, SMIM26 helps maintain the assembly of the mitochondrial translation complex, thereby preserving mitochondrial protein synthesis. This study clearly defines a stepwise regulatory axis in which serine availability influences SMIM26 expression, which in turn regulates mitochondrial translation, thereby modulating complex I activity and ultimately affecting oxidative metabolism. This highlights a direct mechanistic link between a specific nutrient and mitochondrial bioenergetics mediated by a microprotein, which clearly demonstrates that microproteins can function as key elements in the nutrition-metabolism linkage, departing from the nutritional regulatory proteins in existing metabolic regulation studies.

Existing studies on metabolic regulation have focused on kinase activation through nutrient sensing, such as AMPK, or nutrient-responsive transcription factors such as PPAR, ChREBP, and HIF-1. AMPK is a representative energy sensor that is activated by an increase in the intracellular AMP/ADP/ATP ratio. It is activated when nutrient levels are low, and it regulates metabolic pathways, including the stimulation of fatty acid oxidation, glucose uptake, suppression of protein synthesis, and increased autophagy [18]. For example, in skeletal muscles, AMPK activation enhances glucose uptake by facilitating GLUT4 translocation and stimulates mitochondrial biogenesis by activating the PPARγ coactivator 1-alpha (PGC-1α) signaling pathway [53]. PPARα primarily senses fatty acids as endogenous ligands and responds to energy deprivation by upregulating genes involved in fatty acid uptake and β-oxidation within hepatic and muscular tissues [78]. PPARγ plays a pivotal role in adipocyte differentiation and the maintenance of insulin sensitivity; its pharmacological activation by thiazolidinediones enhances glucose utilization and contributes to blood glucose control [19]. In contrast, ChREBP is activated by elevated intracellular glucose or fructose levels, and it governs the transcription of genes associated with glycolysis and de novo lipogenesis. This is a typical nutrient-responsive transcription factor regulatory mechanism that amplifies enzyme gene expression after direct chromatin binding via glucose stimulation [20]. HIF 1 is activated in response to specific nutritional stresses, such as hypoxic conditions or low glucose levels, which regulate the energy supply environment by inducing transcription of glycolytic genes and angiogenic factors [21]. These proteins sense intracellular nutritional status and regulate downstream gene expression at the transcriptional or signaling level to maintain energy homeostasis.

In contrast, SMIM26 exhibits unique ribosome-based regulatory properties. SMIM26 is a very short peptide (approximately 95 amino acids) formed as a microprotein that directly regulates the production of a specific mitochondrial complex I protein (mt ND5) at the translational level in response to changes in serine concentration [22]. This ribosome-based regulatory mechanism operates independently of transcriptional or broader signaling programs and follows a relatively direct route in which the nutrient serine increases SMIM26 expression, which in turn modulates mitochondrial translation, thereby influencing complex I function and oxidative metabolic flux [22]. SMIM26 modulates mitochondrial serine utilization and ND5 mitoribosomal translation under serine-restricted conditions. Current evidence supports SMIM26 as a serine-responsive facilitator of mitochondrial translation rather than as a direct nutrient sensor [22,45,72]. This comparison clearly demonstrates that the SMIM26 study differs from the conventional transcription/signaling-centric regulatory mechanism and establishes a new paradigm in which ribosome-based microproteins functionally interact with nutrition and metabolism (Table 1).

### 2.3. NEMEP and Glucose Uptake During Differentiation

NEMEP is a 63-amino-acid transmembrane microprotein that is derived from a lncRNA, *Gm11549*, which is directly transcribed by nodal signaling [23]. NEMEP is associated with GLUT1 and GLUT3 during mesendodermal differentiation, promoting glucose uptake to sustain the elevated energy demand of this developmental process [54]. Fu *et al*. generated an NEMEP knockout line using mouse embryonic stem cells and demonstrated that NEMEP loss markedly decreased the expression levels of mesendodermal marker genes, glucose uptake, and levels of metabolites involved in glycolysis and the TCA cycle [54]. Consistent with these findings, NEMEP deficiency was shown to impair glucose transporter function, leading to decreased glucose influx. Consequently, intracellular concentrations of key metabolites, such as pyruvate and citrate, were reduced, confirming an overall decline in cellular energy metabolism [54]. Thus, NEMEP represents a paradigm of how microproteins can act at the interface of nutrient sensing and cellular reprogramming, provide immediate and localized control of metabolic fluxes critical for developmental transitions, and highlight that nodal signaling is not simply a transcription factor induction but also directly activates glucose metabolism via microproteins, thereby demonstrating a profound involvement in cellular state and differentiation pathways [5].

## 3. Mechanistic Paradigm Shift

### 3.1. Transcriptional/Post-Translational Control Versus Translational Level

Transcriptional and post-translational regulation represent the fundamental layers of gene expression control, influencing both the production of messenger RNA (mRNA) from DNA and functional state of proteins after synthesis [114]. Transcriptional regulation determines transcript abundance through the activity of transcription factors and chromatin modifications, a process that is inherently slow and often requires hours because it involves changes in mRNA levels followed by translation [115]. Post-translational regulation modifies proteins after they are produced, altering their activity, stability, localization, or interactions through mechanisms such as phosphorylation, acetylation, or ubiquitination [116]. Within the classical model of metabolic control, these regulatory processes are largely mediated by nutrient-sensitive kinases and transcription factors, such as AMPK, PPAR, ChREBP, and HIF-1, which sense cellular nutrient status and initiate signaling cascades or gene expression programs to remodel metabolism [117]. Although these mechanisms are highly effective, they depend on multistep signaling pathways and transcriptional reprogramming; therefore, they generally operate on relatively slow timescales compared with the more immediate modes of translation-level regulation.

In contrast, translational regulation governs gene expression when mRNA is decoded into proteins by ribosomes [117]. As transcripts are already present, this mechanism enables cells to rapidly adjust protein synthesis, often within minutes, in response to environmental or metabolic changes [118]. These findings indicate that microproteins, such as SMIM26, contribute to metabolic adaptation by stabilizing the mitochondrial translation machinery during serine scarcity. However, available evidence does not establish SMIM26 as a primary nutrient sensor, but rather as a factor whose translational function becomes critical under nutrient-limited conditions [22,45,72]. A notable example is the serine-responsive mitochondrial microprotein, SMIM26, which forms a complex with the mitoribosome and serine transporter SFXN1/2 [22]. Through this triad, SMIM26 promotes translation of the ND5 subunit of mitochondrial complex I, thereby sustaining OXPHOS under serine-limiting conditions [22,45]. This form of regulation provides a rapid and localized response to nutrient fluctuations, directly coupling nutrient sensing with mitochondrial function [22]. Collectively, these findings establish microproteins as key players in a previously underappreciated nutrient-regulated axis that is fundamentally distinct from transcription- or signaling-centered paradigms.

### 3.2. Ribosome-Centered Nutrient Sensing Mediated by Microproteins

Ribosomes have long been regarded as cellular organelles responsible for protein synthesis. However, emerging research has revealed that ribosomes are not merely passive translation machines but also dynamic regulators that integrate nutrient availability with translational control [119]. This broader perspective has reshaped our understanding of ribosomes as active hubs of cellular homeostasis.

One of the main findings supporting these theories is the heterogeneity of ribosomes, which indicates that not all ribosomes are identical [120]. Because protein and RNA compositions can vary, a subpopulation of ribosomes can be specialized for the translation of specific mRNAs [121]. For example, eukaryotic ribosomes are 80S in size, comprising 18S rRNAs and 40S-sized subunits containing approximately 33 proteins and 60S-sized large bodies containing 28S, 5.8S, and 5S rRNAs and approximately 47 proteins [121]. This structural complexity supports the function of ribosomes in precisely modulating translation in response to cell signaling, stress, and nutrient availability [122]. This diversity underscores the specialized regulatory functions that ribosomes perform beyond protein synthesis.

Another regulatory feature is protein translational pausing. Translational pausing is a crucial regulatory feature of protein synthesis. Ribosomes frequently pause at specific codons or under defined physiological conditions, a phenomenon increasingly recognized as a programmed strategy rather than a translation error [123]. Recent studies have shown that codon-specific ribosome pausing is regulated by nutrient availability and stress, linking the metabolic status to translation efficiency. For example, arginine depletion induces ribosomal pausing in arginine codons, ultimately leading to decreased rates of protein synthesis [124]. Conversely, amino acid starvation induces ribosome pausing, thereby activating the ZAKα stress response pathway and thus reprogramming metabolism [125]. ZAK (MAP3K20) is a member of the MAP3K family of proteins that is involved in sensing cellular stress. When ribosomes stagnate or collide with each other due to situations, such as amino acid deficiency, inhibition of translation by antibiotics or toxins, mRNA damage, and ribosome collisions due to codon problems, ZAKα detects them directly [125]. When ZAKα is activated, both the p38 mitogen-activated protein kinase and c-Jun N-terminal kinase signaling cascades are stimulated, triggering cellular stress responses and suppressing global protein synthesis [126]. In other words, the ZAKα pathway shows that ribosomes act as sensors to detect nutrient states and stress signals, demonstrating direct involvement in cellular metabolism and stress responses beyond the organelles where protein synthesis occurs [127].

Furthermore, nutrient-dependent pause regulation has been observed during differentiation, most notably during beige adipocyte formation, where ribosomes exhibit increased stalling in glutamate codons [128]. This translational pause is linked to metabolic reprogramming, in which intracellular glutamate is diverted toward glutamine synthesis, thereby reducing charged glutamyl-tRNAs and selectively attenuating the translation of glutamate codon-rich genes, including those involved in actin cytoskeleton organization [128]. As a result, protein synthesis and mRNA stability for these genes decline, illustrating how glutamate-sensitive ribosome pausing integrates the metabolic state with translational control to affect adipocyte differentiation [128]. Thus, ribosomal pausing allows cells to adjust their protein production to meet their metabolic demands. Conversely, when nutrients are limited, ribosomes slow their elongation to conserve energy, whereas when nutrients are abundant, they promote more active translation. These findings, along with ribosome heterogeneity, demonstrate that translational pauses sense nutrient status and reshape cellular metabolism.

Microproteins are a class of small peptides that are translated from both canonical and non-canonical ORFs and have been identified as precise modulators of ribosomal function. By directly interacting with ribosomal subunits or organelles, such as the mitochondria, these microproteins enable ribosomes to control not only which proteins are synthesized but also the rate of their production. For example, the mitochondrial elongation factor 1 microprotein associates with mitoribosomes to regulate mitochondrial translation and respiratory capacity [129]. Similarly, SMIM26 binds to mitoribosomes during serine depletion to preserve mitochondrial respiration [22]. Other microproteins function beyond ribosomal regulation. For example, NEMEP, which is upregulated by nodal signaling, is associated with glucose transporters, such as GLUT1 and GLUT3, to enhance glucose uptake during mesendodermal differentiation, thereby directly linking developmental signaling to cellular metabolic flux [23]. MTLN, another microprotein, is localized to the mitochondrial membrane where it associates with carnitine palmitoyltransferase 1 B (CPT1B) and CYB5B to modulate fatty acid oxidation, thereby coupling ribosome-dependent translation to lipid metabolic processes [48].

Taken together, these findings establish microproteins as metabolic sentinels that empower ribosomes and their associated complexes to sense nutrient cues and reprogram translational and metabolic pathways. By providing a rapid, ribosome-centered regulatory mechanism, microproteins allow cells to adapt swiftly to changing nutrient conditions than possible through transcriptional or post-translational regulation alone.

## 4. Integration with Classical Nutrient Pathways

### 4.1. Comparison of the AMPK–PPAR Axis and Microprotein-Mediated Translation

An easy way to understand the correlation between metabolic pathways is to understand metabolic changes in the fed–fast cycle [130]. Shortly after meal consumption, caloric input may exceed basal needs by >100-fold, whereas during the ensuing fasting interval, stored substrates must sustain energy demands [131]. Maintenance of glucose homeostasis during these transitions is critical because glucose constitutes the principal metabolic fuel for many tissues, particularly the central nervous system [132]. To control this balance, a complex interplay between nutrient sensors and transcriptional regulators coordinates substrate fluxes across organs. Among these, the AMPK–PPAR axis exemplifies the central paradigm of nutrient-sensing biology, mediating both acute and chronic metabolic adjustments [117,133].

The metabolic response can be understood as a temporal sequence with approximate phase lengths varying with meal size, composition, activity, and individual metabolism [130].

(1) Fed state (0–3 h post-meal): Nutrient absorption, insulin secretion, and glycogen/lipid storage are predominant.
(2) Post-absorptive/Early fasting (~3–12/18 h): Hepatic glycogenolysis maintains blood glucose.
(3) Fasting (~18–48 h): Glycogen depletion shifts the reliance on adipose lipolysis and hepatic gluconeogenesis.
(4) Starvation (days/weeks): Ketogenesis provides alternative fuels, sparing glucose for obligate tissues such as those of the central nervous system.

This section elaborates on the definition and molecular characteristics of each stage of the feed-fast cycle by integrating the temporal dynamics of AMPK, PPARs, and the emerging microprotein-mediated translational control.

#### 4.1.1. AMPK–PPAR Axis in the Fed–Fast Cycle

- Fed state (0–3 h postprandial)

In the immediate postprandial state, nutrient availability is abundant and circulating glucose and insulin concentrations increase sharply. Elevated ATP/AMP ratios inhibit AMPK activity, thereby suppressing catabolic signaling [117]. Instead, anabolic pathways predominate, promoting glycogen storage, de novo lipogenesis, and protein synthesis primarily through activation of the mTORC1 [134]. Concurrently, hepatic PPARα activity decreases, thereby reducing fatty acid oxidation, while PPARγ promotes lipid storage and adipogenesis [135]. Thus, the fed state is defined by substrate storage and nutrient assimilation. Over the ensuing hours, this transcriptional skew consolidates the storage phenotype, with hepatic glycogen and lipid synthesis, adipose triacylglycerol accumulation, and muscle glycogen repletion as the net outcomes [117].

- Early fasting state (3–12/18 h postprandial)

As insulin levels decrease and glucagon levels increase, glycogenolysis becomes the primary source of circulating glucose. Cellular energy charge declines, leading to AMPK activation [136]. Activated AMPK phosphorylates acetyl-CoA carboxylase, leading to reduced malonyl-CoA levels and consequent relief from CPT1 inhibition, thereby promoting fatty acid oxidation [137]. Simultaneously, AMPK promotes GLUT4 translocation in skeletal muscles and inhibits mTORC1 [133]. PPARα expression begins to rise in the liver, reinforcing fatty acid oxidation, while PPARδ supports oxidative metabolism in muscles [138].

On a slower timescale (hours), PPARα mRNA and protein levels in the liver are induced on an empty stomach, triggering upregulation of liver genes, such as *Cpt1a*, *Acadm*, and *Acox1*, that mediate fatty acid uptake and β-oxidation, thereby sustaining mitochondrial fatty acid catabolism under reduced glucose supply conditions [139]. Simultaneously, in skeletal muscle, PPARδ promotes transcription of oxidative metabolic genes, such as *Cpt1b* and *Pdk4*, and promotes the oxidative capacity of mitochondria and transition to a more oxidative (type 1) fibrotic phenotype [140]. Although these classical nutrient-based transcriptional programs operate within hours, they highlight the temporal hierarchy between rapid post-transcriptional coordination and slow genome response, in contrast to novel microprotein-mediated translational regulation, which can regulate metabolic flow within minutes.

- Fasting state (18–48 h)

During a fasting state (18–48 h), AMPK remains acutely activated, rapidly phosphorylating downstream targets to suppress anabolic processes, stimulate autophagy, and initiate mitochondrial biogenesis via PGC-1α coactivation in parallel. Nuclear receptors, such as PPARα and PPARγ, engage more slowly, requiring hours to induce transcriptional reprogramming of β-oxidation, ketogenesis, and adipose lipolysis [141]. This temporal distinction highlights a fundamental principle: classical nutrient regulators act on different time scales, with AMPK providing immediate (seconds to minutes) signaling control, whereas transcription factor-mediated programs unfold over multi-hour to day-long periods [142]. Compared with emerging microprotein-mediated translation, which can directly adjust mitochondrial or transporter function within minutes, classical pathways are inherently slower because they depend on chromatin binding, mRNA synthesis, and protein turnover [55,129,143].

- Starvation (days to weeks)

During starvation (days to weeks), AMPK activity remains persistently elevated, enforcing an acute energy-conserving setpoint across tissues [117]. Meanwhile, nuclear receptors, such as PPARα and PPARδ, sustain long-term transcriptional programs that drive fatty acid oxidation and ketogenesis, and PPARγ maintains adipocyte function [141]. These transcriptional adaptations unfold slowly, often requiring days, because they depend on chromatin remodeling, mRNA synthesis, and protein turnover [142]. In contrast, emerging evidence has shown that microprotein-mediated translational control can modulate mitochondrial respiration or nutrient transport within minutes, independent of slower transcriptional cascades. These differences highlight the underlying temporal differences. Classical nutrient regulators progressively readjust metabolism via gene expression remodeling, whereas microproteins act as fast-acting metabolic switches, complementing slow and persistent reprogramming driven by the AMPK–PPAR pathway without replacing it.

#### 4.1.2. Temporal Dynamics of the AMPK–PPAR Axis and Microprotein

**Figure 2:**
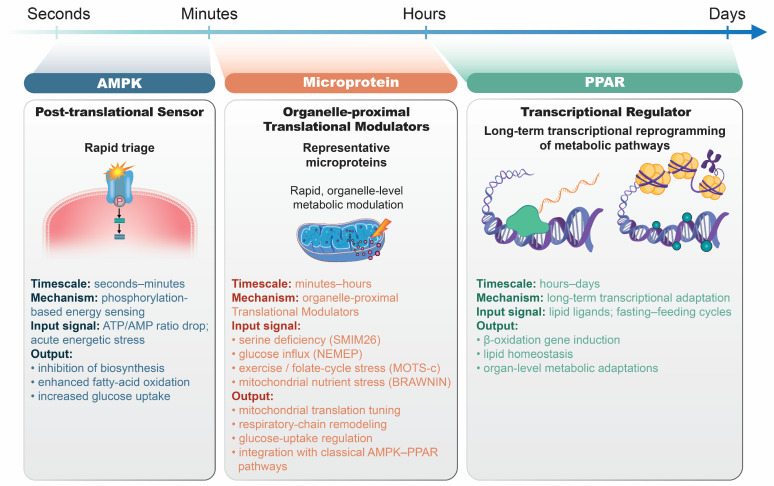
Temporal dynamics of nutrient regulation by AMPK, microproteins, and PPAR. Arrows indicate regulatory directionality. Colors distinguish the three major classes of metabolic regulators (AMPK: post-translational sensor in blue; microproteins: organelle-proximal modulators in orange/blue; PPAR: transcriptional regulators in green/purple). Shapes represent conceptual protein complexes and DNA structures.

The temporal dynamics of the AMPK–PPAR axis illustrate why classical nutrient regulation often unfolds slowly. AMPK acts within seconds to minutes through post-translational phosphorylation, providing a rapid metabolic triage under energy stress. In parallel, PPARs reshape gene expression via transcriptional programs, a process that inherently requires hours to days because it depends on chromatin remodeling, mRNA synthesis, and protein turnover. Since both layers operate together, full metabolic reprogramming is gradual, ensuring durable adaptation, but at the cost of temporal delay. In contrast, microprotein-mediated translation operates faster than transcriptional responses, thereby providing complementary rapid adjustments that directly modulate mitochondrial activity or nutrient transport within minutes. This sharp difference reveals the complementary roles of classical regulators as slow but enduring remodelers versus microproteins as fast-acting switches that fine-tune energy metabolism in real time (Figure 2).

AMP-activated protein kinase (AMPK) responds within seconds to minutes via post-translational phosphorylation, enabling rapid triage under acute energy stress. Peroxisome proliferator-activated receptors (PPARs) act on a slower timescale, from hours to days, through transcriptional remodeling that supports long-term adaptation. In contrast, microproteins function directly at the translational or organelle level, adjusting mitochondrial activity or nutrient transport within minutes, in a manner that does not require slower transcriptional responses. Together, these regulators establish a layered nutrient-sensing framework in which AMPK provides immediate responses, PPARs coordinate durable remodeling, and microproteins serve as fast-acting translational modulators that complement classical pathways. Arrows indicate regulatory directionality. Colors distinguish the three major classes of metabolic regulators (AMPK: post-translational sensor in blue; microproteins: organelle-proximal modulators in orange/blue; PPAR: transcriptional regulators in green/purple). Shapes represent conceptual protein complexes and DNA structures.

#### 4.1.3. Comparison with Microprotein-Mediated Translational Control

Recent studies have identified microproteins, which are peptides encoded by sORFs, as additional regulators of nutrient-responsive metabolism. For example, SMIM26 responds to serine availability by binding to mitochondrial transporters and mitoribosomes, thereby directly promoting the complex I subunit, ND5, and preserving OXPHOS [22]. Such mechanisms operate at the translational level, enabling adjustments within minutes to tens of minutes, faster than transcription-dependent pathways, but slower than the immediate phosphorylation events mediated by AMPK [71]. Another example is MOTS-c, a mitochondria-derived peptide that activates AMPK and modulates PPAR signaling, thereby bridging microprotein action with classical pathways [24].

Taken together, AMPK provides rapid energy state sensing, PPARs confer durable transcriptional remodeling, and microproteins offer an intermediate timescale for direct translational control (Table 2). This layered regulation ensures flexibility and resilience of the metabolic network across fed–fast transitions. An expanded comparative overview of these temporal and mechanistic layers, including the distinct onset kinetics and regulatory modalities of AMPK, PPAR, and microprotein-mediated pathways, is summarized in Table 3, offering a broader framework for understanding how nutrient-responsive regulators coordinate metabolic adaptation.

### 4.2. Crosstalk Between Nutrient Transporters, Mitochondria, and Ribosomes

Recent studies have identified microproteins as molecular bridges that coordinate nutrient transport, mitochondrial bioenergetics, and ribosomal translation. A representative example is the serine-responsive mitochondrial microprotein SMIM26, which associates with the serine transporters SFXN1/2 to maintain translation of the complex I subunit, ND5, thereby supporting OXPHOS during amino acid limitation [22]. Similarly, the nodal-induced microprotein, NEMEP, directly binds to GLUT1 and GLUT3, thereby enhancing glucose uptake during mesendodermal differentiation. Through this interaction, NEMEP links nutrient influx to cell fate programs by increasing glycolytic flux and TCA cycle intermediates [23]. Another example is MTLN, which localizes to the outer mitochondrial membrane and associates with CPT1B and CYB5B, thereby precisely modulating fatty acid β-oxidation and respiratory activity [48].

Collectively, these findings illustrate that nutrient transporters, mitochondria, and ribosomes are tightly integrated via microprotein-mediated mechanisms. In addition to their canonical role in mRNA decoding, ribosomes also function as nutritionally responsive hubs. For example, ribosomal subunits or microproteins associated with the mitoribosome can modulate translational output according to the availability of amino acids or energy [55]. Pausing translation in certain codons further integrates nutritional status into ribosomal dynamics, balancing energy-consuming protein synthesis with metabolic supply [144]. Microproteins act as sentinels that couple transporter activity and mitochondrial metabolism with ribosomal translation. Together, these interactions establish a rapid, localized nutrient-sensing axis, in which transporters deliver substrates, mitochondria regulate bioenergetic output, and ribosomes modulate protein synthesis. By circumventing the requirement for extensive transcriptional reprogramming, the coordinated actions of nutrient transporters, mitochondria, and ribosomes enable cells to implement rapid and localized metabolic adjustments [23]. Such translational and organelle-level regulation provides an efficient alternative to transcription factor-driven remodeling, which typically operates on slower timescales due to chromatin restructuring, RNA synthesis, and protein turnover [48]. Consequently, this tripartite mechanism strengthens cellular resilience during developmental transitions and under metabolic stress, allowing nutrient fluctuations to be buffered at the level of translation and bioenergetics rather than relying exclusively on delayed transcriptional programs [22,73].

## 5. Physiological and Pathological Implications of Nutrient-Sensing Microproteins

### 5.1. Microproteins in Obesity and Energy Homeostasis

Adipose-derived microproteins contribute to the regulation of the energy balance and obesity. The *Gm8773* gene produces a secreted peptide homologous to human *FAM237B*, which is predominantly expressed in neurons of the hypothalamic arcuate nucleus [108]. Administration of recombinant mouse *FAM237B* into the cerebral ventricles of mice with diet-induced obesity resulted in a significant increase in food consumption, suggesting that this peptide functions as a central orexigenic signal within the brain–adipose regulatory network [25]. Another microprotein, *MICT1*, encoded by *C16orf74*, is selectively expressed in BAT and markedly upregulated in response to cold stimulation. At the molecular level, *MICT1* binds to protein phosphatase 2B (PP2B, also known as calcineurin) via a conserved docking sequence (PNIIIT), which suppresses PP2B-dependent dephosphorylation of the protein kinase A (PKA) regulatory subunit IIβ (RIIβ). This inhibition maintains elevated PKA activity, leading to the activation of thermogenic transcriptional programs and increased mitochondrial oxygen consumption [107]. In vivo studies have demonstrated that *MICT1* overexpression specifically in BAT enhances whole-body energy expenditure and confers protection against diet-induced and genetic forms of obesity, as well as insulin resistance. Conversely, the targeted deletion of *MICT1* in BAT diminishes thermogenic efficiency, resulting in greater adiposity and metabolic impairment. Collectively, these observations identify *MICT1* as a potential molecular target for therapeutic interventions in metabolic diseases [107].

Additionally, microproteins that modulate mitochondrial ATP-sensitive potassium channels (MitoK_ATP) appear to regulate BAT differentiation and thermogenesis. Genetic deletion of the pore-forming subunits of MitoK_ATP in human pre-adipocytes impairs cellular respiration and differentiation into mature adipocytes. Conversely, pharmacological inhibition of MitoK_ATP in mature brown adipocytes enhances β_3_-adrenergic-stimulated oxygen consumption, suggesting that targeting this pathway may amplify thermogenic responses and combat obesity [75].

Together, these findings provide compelling evidence that microproteins expressed in adipose tissue can modulate central and peripheral mechanisms governing energy expenditure, feeding behavior, and thermogenesis, and offer new molecular targets for anti-obesity therapies.

### 5.2. Therapeutic Potential of Microproteins in Type 2 Diabetes Mellitus (T2DM)

T2DM arises from the combined effects of impaired insulin signaling, β-cell failure, and chronic inflammatory activation, with mitochondrial and endoplasmic reticulum stress, as well as lipid overload, serving as key molecular drivers of disease development [76,77]. Microproteins, which are small peptides comprising < 150 amino acids, have recently been recognized as key regulators of metabolic homeostasis [52,145]. These molecules regulate multiple aspects of cellular metabolism, such as glucose uptake, mitochondrial respiration, lipid oxidation, and stress adaptation, and are potential targets for metabolic therapy. In this section, we summarize key microproteins implicated in glucose and insulin regulation and discuss their potential translational applications in diabetes management.

#### 5.2.1. Microproteins Implicated in Glucose and Insulin Regulation

##### MOTS-c

MOTS-c, previously introduced in Section 3, is a short peptide encoded within the mitochondrial genome, comprising 16 amino acids, and exerts metabolic effects by activating AMPK and enhancing GLUT4-mediated glucose uptake [56,87]. Specifically, it activates AMPK, which acts as an energy sensor within the cells, and GLUT4, which facilitates cellular glucose uptake. In murine models, treatment with MOTS-c improves insulin responsiveness, counteracts obesity induced by high-fat feeding, and reduces β-cell damage driven by autoimmune mechanisms via regulation of mTORC1 activity [87,88]. As we know, in some types of diabetes (particularly type 1 diabetes and some autoimmune β-cell damage), immune cells attack the insulin-producing cells (β-cells) of the pancreas. If mTORC1 is overactive, immune cells can become overactive and attack β-cells [88]. However, MOTS-c functions as a negative regulator of mTORC1 activity, thereby attenuating excessive immune activation and protecting pancreatic β-cells from immune-mediated injury [89]. These diverse effects suggest potential interventions for both T2DM and diabetes.

##### Adrenomedullins (ADMs)

Surprisingly, a recent investigation by Cho *et al.* revealed that increased serum levels of ADM in obesity attenuate insulin signaling through enhanced dephosphorylation of the insulin receptor within vascular endothelial cells [109]. Specifically, ADM activates protein tyrosine kinase 1 B (PTP1B) via stimulatory G protein (Gs), a heterotrimeric GTP-binding protein that couples G protein-coupled receptors to adenylyl cyclase and stimulates cyclic AMP (cAMP) production, and the downstream PKA pathway [110]. Subsequently, PTP1B dephosphorylates key tyrosine residues of the insulin receptor, thereby attenuating insulin signaling. Notably, patients with obesity with an endothelial cell-specific deletion of the ADM receptor display improved insulin sensitivity, which was accompanied by enhanced insulin-stimulated endothelial nitric oxide synthase (eNOS) phosphorylation and increased skeletal muscle perfusion [109]. Furthermore, administration of the ADM receptor antagonist (24–50) in obese mice restored systemic insulin sensitivity. These findings, unlike those of previous studies that primarily focused on systemic insulin resistance or adipose tissue dysfunction, provide a novel therapeutic target by first demonstrating that blocking the ADM receptor, a microprotein in vascular endothelial cells, restores insulin responsiveness and improves glucose homeostasis [109].

ADM2 is initially produced as a 148-amino acid prepropeptide that contains a signal sequence, prohormone region, and bioactive mature peptide. Upon secretion, ADM2 interacts with the calcitonin receptor-like receptor in a complex with receptor activity-modifying protein 2 (RAMP2) or RAMP3. This receptor engagement activates the guanine nucleotide-binding protein Gs alpha subunit (Gαs), which in turn stimulates adenylate cyclase activity, leading to elevated intracellular cAMP concentrations [111,112]. Through this signaling cascade, ADM2 promotes vascular smooth muscle relaxation, enhances nutrient perfusion to peripheral tissues, and contributes to the regulation of systemic energy metabolism. Moreover, ADM2 expression has been detected in metabolically active tissues, including the adipose tissue, skeletal muscle, pancreas, and vascular endothelium, indicating its physiological role in coordinating metabolic homeostasis [109]. The ability of ADM2 to activate cAMP and its downstream PKA signaling confers vasodilatory and metabolic regulatory properties. Given the overlap of vascular and metabolic dysfunction in T2DM, ADM2 is a promising candidate for biomarker and therapeutic development [109,112]. Moreover, Li *et al*. demonstrated that ADM2 improves insulin resistance and reduces obesity in mice by promoting vasodilation and microvascular perfusion, thereby increasing glucose uptake in skeletal muscles [113]. In skeletal muscle and adipose tissue, ADM2 activates cAMP/PKA and AMPK crosstalk to stimulate GLUT4 membrane translocation and exerts anti-inflammatory effects that improve insulin resistance [113]. Overall, evidence indicates that ADM2 exerts diverse physiological effects that enhance glucose regulation.

##### Mitoregulin

Mitochondrial dysfunction is a hallmark of T2DM, which leads to impaired fatty acid β-oxidation, accumulation of lipotoxic intermediates, oxidative stress, and reduced metabolic flexibility in insulin-responsive tissues [79,146]. Among the mitochondrial microproteins involved in maintaining metabolic balance, MTLN, encoded by the *LINC00116* locus and introduced in Section 2, plays a supportive role in sustaining mitochondrial lipid homeostasis and respiratory efficiency [147]. Located mainly in the outer mitochondrial membrane, MTLN interacts with key lipid-metabolizing enzymes, such as CPT1B, to facilitate the processing of very-long-chain fatty acids [48]. Loss of MTLN impairs OXPHOS, promotes lipid accumulation, and alters the mitochondrial stress response, all of which are well-known contributors to the pathogenesis of T2DM [48,80]. Notably, the metabolic consequences of MTLN deficiency may vary, depending on the context, and certain dietary settings may mitigate rather than exacerbate insulin resistance [48]. MTLN may attenuate lipid-driven insulin resistance and systemic inflammation by preserving mitochondrial membrane integrity, optimizing β-oxidation, and reducing reactive oxygen species production, suggesting its potential utility in re-establishing mitochondrial function and promoting metabolic flexibility in T2DM [48,79,80].

##### HN

The mitochondria-derived peptide HN, comprising 24 amino acids, confers protection against cellular stress by exerting cytoprotective and anti-apoptotic effects, and has been implicated in the development and treatment of T2DM [90]. Preclinical in vitro studies have demonstrated that HN and its analogs protect pancreatic β-cells from cytokine-induced apoptosis, enhance glucose-stimulated insulin secretion, and promote mitochondrial biogenesis, AMPK activation, and ATP production in β-cells, which are mechanisms central to preserving insulin production and signaling under glucotoxic conditions [90,91]. In rodent models, treatment with HN or its analogs enhances insulin action, decreases blood glucose levels, and prevents early diabetes progression, suggesting its utility as a therapeutic candidate [92]. Clinically, altered circulating HN levels have been reported in patients with prediabetes, T2DM, gestational diabetes, and type 1 diabetes, suggesting that HN may serve as a biomarker of metabolic dysfunction compensatory response to oxidative stress and mitochondrial impairment [90,92]. Collectively, these findings indicate that HN enhances insulin responsiveness, maintains β-cell integrity, and alleviates mitochondrial stress, underscoring its dual value as a diagnostic biomarker and therapeutic candidate for diabetes.

##### BRAWNIN

BRAWNIN is a 71-amino acid microprotein that is localized to the inner mitochondrial membrane and encoded by the *C12orf73* gene. It plays an indispensable role in the assembly of respiratory chain complex III within the mitochondrial electron transport system [49]. Loss of BRAWNIN disrupts complex III formation, resulting in impaired OXPHOS and decreased ATP synthesis [49].

BRAWNIN expression is tightly controlled by the cellular energy-responsive AMPK pathway and increases upon nutritional stress, including glucose or amino acid deficiency, or pharmacological AMPK activation, such as treatment with 5-aminomidazole-4-carboxamide ribonucleotide (AICAR) [49]. AICAR is an AMP mimetic that acts as an AMP within cells and activates AMPK [93]. AICAR activates AMPK signaling by simulating an intracellular energy-deficient condition, and is frequently used as an experimental model to assess transcriptional responses of energy metabolism genes such as *BRAWNIN* [5,93].

Nutrient stress, defined as a state of nutrient insufficiency or imbalance, triggers AMPK activation to restore energy homeostasis by enhancing mitochondrial efficiency through factors such as BRAWNIN [93]. Moreover, an increase in BRAWNIN protein levels has been observed under AICAR (AMPK activator) treatment or during glucose, serum, and fatty acid starvation conditions [49]. In contrast, when *BRAWNIN* was knocked down in U87MG glioma cells by small interfering RNA or short hairpin RNA transfection, a marked reduction in mitochondrial respiratory parameters and ATP production was observed [49]. This suggests that BRAWNIN is essential for mitochondrial energy production, implying that decreased BRAWNIN impairs the cellular ability to maintain energy balance [5,49,84].

T2DM is closely associated with impaired mitochondrial function and reduced OXPHOS efficiency, primarily in skeletal muscle and cardiac tissue, leading to insulin resistance and impaired glycation (fatty acid and glucose oxidation) [85]. In this context, the pivotal role of BRAWNIN in complex III assembly and OXPHOS maintenance suggests that BRAWNIN activation may be a potential strategy to restore mitochondrial energy metabolism and improve insulin sensitivity [84].

These insights suggest that BRAWNIN is a promising therapeutic regulator for restoring mitochondrial oxidative capacity and improving systemic metabolic health in diabetes. Nutritional- or gene-based strategies to enhance the stability or function of BRAWNIN may mitigate mitochondrial bioenergetic failure and its downstream metabolic consequences. Although no nutrients have been identified to directly regulate BRAWNIN to date, it is possible that there is indirect regulation through the AMPK activation pathway via mixed polyphenols and micronutrients [86].

##### Mitolamban (Mtlbn)

Mtlbn, a microprotein comprising approximately 47 amino acids predominantly expressed in cardiac tissue, is localized to the inner mitochondrial membrane, where it facilitates the organization of complex III and its super-complex structures [81]. In *Mtlbn* knockout mice, impaired complex III biogenesis is associated with disordered metabolic enzyme function and global metabolic dysregulation, consistent with the phenotype of complex III deficiency [81]. In contrast, mice with cardiac-specific overexpression (transgenic mice) display cardiomyopathy characterized by histological remodeling, mitochondrial structural defects, and increased oxidative stress [81].

Although this study did not use a diabetic model directly, the findings are relevant to the context of diabetic metabolic pathophysiology. In T2DM, both β-cell dysfunction and insulin resistance are closely associated with mitochondrial dysfunction, which is recognized as the central pathological mechanism [82]. The suppression of complex III activity, together with altered intermediary metabolism in *Mtlbn* knockout mice, may reflect a metabolic phenotype analogous to that of diabetes. Additionally, the increased oxidative stress observed in transgenic mice parallels mitochondrial inflammation and oxidative damage frequently reported in diabetic hearts [83]. Oxidative imbalance is a well-established cause of diabetic cardiomyopathy.

More broadly, emerging evidence shows that microproteins play critical roles in metabolic regulation, mitochondrial function, and nutrient signaling [57,148]. Mtlbn is a fine-tuner of the electron transport chain and a potential therapeutic target for metabolic disorders, such as diabetes-related cardiac disease, where mitochondrial efficiency and redox balance are impaired. Although direct nutrient regulation of Mtlbn has not been shown, factors influencing mitochondrial function and complex III activity, such as AMPK modulators including resveratrol, metformin-like agents, and exercise, may enhance mitochondrial function, thereby indirectly modulating Mtlbn expression or activity.

##### PIGBOS

PIGBOS is a 54-amino acid mitochondrial microprotein localized at contact sites between the ER and mitochondria, where it modulates the unfolded protein response (UPR), a signaling pathway associated with ER stress [58]. The UPR is triggered by the accumulation of misfolded proteins in the ER, and functions to reestablish protein-folding equilibrium. Under metabolic stress conditions, such as nutrient overload, inflammation, and oxidative injury, sustained UPR activation impairs insulin activity and contributes to metabolic dysregulation [104].

PIGBOS enhances ER-stress signaling and apoptosis, a maladaptive state known to drive metabolic pathology [105]. In diabetes, chronic ER stress and sustained UPR activation impair insulin signaling in the liver and adipose tissue, worsen hepatic steatosis, and compromise pancreatic β-cell function, which are the core mechanisms of insulin resistance and T2DM [105]. PIGBOS may help preserve insulin sensitivity and β-cell viability by constraining excessive UPR activity [105]. Thus, PIGBOS and related mitochondrial–ER interface microproteins are promising modulators and potential therapeutic targets for the restoration of metabolic control in diabetes.

#### 5.2.2. Translational and Clinical Applications in Diabetes Management

Microproteins offer several therapeutic benefits in the management of diabetes. A prime example is the multi-targeting effects, which align with the multifactorial nature of diabetes [56]. For example, MOTS-c exerts multiple effects on glucose uptake, inflammatory signaling, and lipid metabolism [24]. Notably, the small size of microproteins facilitates tissue penetration and may allow them to cross the blood–brain barrier, as demonstrated by MP31 in cancer models [149]. As microproteins are endogenous peptides, they may have a lower risk of immunogenicity than that of fully synthetic formulations [149,150]. Furthermore, some microproteins have potential as biomarkers. For example, circulating ADM2 could guide patient selection and dosing, thereby helping personalize treatment [112]. Clinically realizing these benefits requires systematic optimization of safety, delivery, and dosing, as well as mechanistic and efficacy studies in human models [151].

### 5.3. Microproteins in Non-Alcoholic Fatty Liver Disease (NAFLD) and Lipid Metabolism

A comprehensive proteo-transcriptomic framework of NAFLD progression was recently constructed in a metabolism study by Govaere *et al*., in which 4730 circulating proteins were profiled from 306 histologically characterized patients and correlated with the hepatic transcriptome [152]. A panel of 31 proteo-transcriptomic markers that distinguished active steatohepatitis and advanced fibrosis was identified, and novel circulating biomarkers and insights into the cellular origins of these signatures were offered through single-cell deconvolution [152]. Although these data primarily point to classical proteins as indicators of NAFLD and its severe manifestation, non-alcoholic steatohepatitis (NASH), to date, most microprotein studies have concentrated on mitochondrial function [153], ribosome regulation [154], or immune signaling [5,155]. However, emerging evidence that microproteins affect metabolic diseases highlights their potential as therapeutic agents.

### 5.4. Stress-Responsive Microproteins and Cellular Protection

A recent study discovered a special splice variant of ubiquitin fusion degradation 1 (UFD1) that forms a microprotein called UFD1s instead of the usual UFD1f protein [106]. Unlike UFD1f, UFD1s interacts with the E3 ubiquitin ligase MARCH7. Through this interaction, it competes with UFD1f and reduces K63-linked ubiquitination, demonstrating that UFD1s directly participate in regulating protein ubiquitination under stress. These findings highlight that UFD1s are regulatory microproteins involved in the modulation of protein ubiquitination during cellular stress responses [106].

#### 5.4.1. Ubiquitination Basics

Ubiquitin, a 76-amino acid protein, functions as a molecular modifier that is covalently attached to other proteins to regulate their stability and signaling [156]. Ubiquitination is a reversible post-translational modification mediated by E3 ligases and reversed by deubiquitinating enzymes (DUBs). E3 ligases, such as tripartite motif-containing protein 8 and TNF receptor-associated factor 6, facilitate ubiquitin conjugation to specific substrates, leading to enhanced hepatic steatosis, inflammation, and fibrosis [157]. In contrast, DUBs, such as ubiquitin-specific peptidase 4 and OTU domain-containing ubiquitin aldehyde-binding protein 1, remove these chains, stabilize proteins, and often exhibit protective effects against liver damage [158]. Previous studies on NAFLD and NASH primarily focused on these key enzymes [156,159].

#### 5.4.2. Why UFD1s Is Different

Remarkably, the recent discovery of UFD1, a microprotein encoded by a splice variant, opened a new chapter in ubiquitin-based regulation. Unlike conventional enzymes, UFD1 regulates ubiquitination indirectly. It competes with the E3 ligase MARCH7, reduces the K63-linked ubiquitination of UFD1f, and destabilizes inositol polyphosphate multikinase via K48- and K11-linked chains [106]. Together, these changes activate two protective processes: autophagy, which functions in cellular recycling and cleanup, and fatty acid oxidation, which allows cells to burn fat for energy in the mitochondria. Both mechanisms are essential for protecting the liver cells against metabolic stress. In animal models, the loss of UFD1 exacerbates metabolic dysfunction and accelerates NASH progression. Conversely, re-administration of UFD1 via plasmid DNA or circular RNA delivery alleviated NASH pathology, demonstrating the protective role of this microprotein in vivo [106]. This study is the first to demonstrate that a microprotein can reshape liver metabolism through the ubiquitin pathway, which expands the field beyond conventional enzymes such as E3 ligases and DUBs, and highlights microproteins as new therapeutic targets for metabolic diseases [106].

## 6. Diet-Responsive Microproteins and Nutritional Regulation

Microproteins constitute a nutrient-sensing layer that couples dietary inputs with cellular energetics, endocrine signaling, and organismal metabolism. Recent findings have demonstrated that these molecules operate in multiple metabolic organs, including the adipose tissue, muscle, liver, and brain, providing new insights into precision nutrition and metabolic health interventions.

### 6.1. Adaptive Microproteins Governing Brown Adipose Thermogenesis

Increasing evidence suggests that these molecules participate in nutrient-sensing pathways that link dietary inputs to energy homeostasis. In particular, diet-responsive microproteins in brown and white adipose tissues represent a novel axis for understanding thermogenesis and metabolic disease susceptibility.

Microproteins play an essential role in coordinating thermogenic responses within BAT by integrating metabolic signals [160]. In this context, MICT1, a microprotein encoded by *C16orf74*, is highly expressed in brown adipocytes and is rapidly induced by exposure to cold, establishing it as an adaptive microprotein that senses metabolic demands [50]. Mechanistically, MICT1 binds to PP2B (calcineurin) via its PNIIIT motif, preventing dephosphorylation of the PKA regulatory subunit, RIIβ, and maintaining PKA activity in brown adipocytes, which amplifies β_3_-adrenaline-stimulated thermogenic gene expression and oxygen consumption [50]. In vivo, MICT1 deletion in uncoupling protein 1-positive cells diminishes the thermogenic capacity of BAT, leading to obesity and insulin resistance, whereas MICT1 overexpression, blocking its binding to PP2B, augments energy expenditure and confers resistance to diet-induced metabolic dysfunction [107]. From a nutritional perspective, dietary thermogenic compounds, such as caffeine, catechins, and capsaicin, which are known to stimulate BAT activity, could synergize with MICT1-mediated pathways to enhance energy expenditure [161,162]. Moreover, the nutrient-responsive regulation of sORF-encoded microproteins suggests that various dietary patterns, such as protein intake, fasting-refeeding cycles, and ketogenic diets, may modulate MICT1 expression and function [5]. These insights support the potential of functional foods or nutraceuticals targeting MICT1 activity, as well as combined diet–drug strategies, to prevent obesity and metabolic diseases through enhanced thermogenesis. Similarly, deletion of Family with Sequence Similarity 210 Member A (*FAM210A*) in BAT aggravates high-fat diet-induced metabolic impairment, underscoring the diet-sensitive regulation of microprotein function [163]. Collectively, these reports suggest that BAT-enriched microproteins act as modulators of diet-induced metabolic adaptations.

### 6.2. Nutritional Targeting of Mitochondrial Microproteins

Mitochondrial microproteins represent an underexplored class of nutrient-sensitive regulators of cellular energetics, each offering a distinct avenue for nutritional targeting. BRAWNIN (*C12orf73*) is indispensable for complex III assembly, and its expression is upregulated via AMPK signaling, suggesting that dietary interventions, such as caloric restriction (CR), intermittent fasting, or polyphenol intake, may preserve OXPHOS capacity [49]. The mitochondrial peptide, *MOTS-c*, comprising 16 amino acids and transcribed from the *12S rRNA* region, plays a regulatory role in the folate–methionine metabolic pathway and enhances insulin sensitivity. Its activity is induced by exercise and nutritional stress, positioning it as a candidate for dietary strategies that mimic exercise or fasting [56]. MICT1 (*C16orf74*) sustains β-adrenergic thermogenic signaling in brown adipocytes by preventing calcineurin-mediated PKA inactivation, raising the possibility that thermogenic dietary compounds, such as caffeine, catechins, or capsaicin, could synergize with its pathway to enhance energy expenditure [107]. Finally, the mitochondria-encoded microprotein, HN, comprising 24 amino acids and translated from a sORF in the *16S rRNA* gene (*MT-RNR2*), was initially characterized in 2001 for its neuroprotective activity against Alzheimer’s disease-linked cellular damage. In this seminal study, Nishimoto *et al*. demonstrated that HN protects neuronal cells from apoptosis induced by amyloid-β exposure, establishing its role as a cytoprotective and anti-apoptotic peptide [31]. A subsequent study has expanded the neuroprotective paradigm of HN to encompass broader stress modalities, such as mitigating oxidative stress and ischemia–reperfusion damage in cardiomyocytes, endothelial cells, and fibroblasts through antioxidant pathway activation [164,165,166]. Moreover, recent reviews confirmed its role in reducing inflammation and mitochondrial stress associated with aging and metabolic decline [94].

Collectively, these examples demonstrate that mitochondrial microproteins not only safeguard bioenergetics and stress responses but also provide actionable entry points for nutritional modulation and functional food design, advancing precision nutrition for metabolic disease prevention.

### 6.3. Caloric Restriction, Fasting, and sORF Expression

Metabolic disorders and aging are closely linked to impaired protein homeostasis and dysregulated nutrient signaling [167,168]. Recent studies have revealed that atypical ORFs generate microproteins, previously overlooked in functional genomics, that play crucial regulatory roles in muscle physiology, metabolism, and endocrine adaptation [169,170,171]. Nutritional interventions, such as CR, protein quality control, and pharmacological mimetics, such as rapamycin, have emerged as powerful tools for restoring homeostasis [172,173,174]. This chapter synthesizes recent findings to highlight how nutritional manipulation can reshape microprotein biology and pave the way for new directions in precision nutrition [175,176].

#### 6.3.1. Caloric Restriction/Rapamycin Remodel Non-Canonical ORF Translation

Notably, CR and rapamycin reprogram the translation of non-canonical ORFs in aged skeletal muscles. To examine the influence of these interventions on proteostatic remodeling, ribosome footprinting was performed on muscle tissues from aged mice maintained on standard diets or subjected to prolonged CR or rapamycin treatment. Notably, CR promotes stop-codon readthrough and enhances the translation of downstream ORFs, whereas rapamycin preferentially restructures translation at uORFs [172]. Proteomic validation confirms the existence of functional microproteins that link dietary restriction to rejuvenated microprotein expression. This provides direct evidence that nutritional interventions can unlock hidden proteomic layers relevant to muscle maintenance [172].

#### 6.3.2. Adropin as a Nutrient-Responsive Microprotein Hormone

Adropin, a 76-amino acid peptide product of the *energy homeostasis-associated* (*ENHO*) gene, is largely expressed in metabolic and vascular tissues, including the liver, brain, and endothelium [95]. Unlike insulin, which is commonly derived from adipocytes or pancreatic β cells, adropin is not restricted to a single organ, but rather integrates signals across multiple tissues [96]. Interestingly, adropin expression is responsive to nutrients, particularly macronutrient composition. For example, a high-fat diet suppresses adropin, whereas carbohydrate intake or CR increases adropin [97]. This metabolic regulatory capacity has been well-characterized in animal models. Liver-specific loss of adropin gene in mice leads to metabolic disturbances characterized by glucose intolerance, systemic insulin resistance, enhanced adiposity, and transcriptional activation of lipogenic pathways in the liver [98]. Conversely, adropin gene upregulation triggers AMPK activation and lipid oxidation, contributing to the metabolic stability of hepatic and adipose tissues [99].

##### CR and Adropin

Recent studies have demonstrated that adropin expression is upregulated under CR conditions, and that this increase is strongly correlated with enhanced lipid metabolic efficiency, improved glucose handling, and greater insulin responsiveness [100]. CR improves mitochondrial efficiency, and NAD-dependent metabolic pathways and adropin appear to interact with these mechanisms to suppress the development of age-related metabolic diseases [99]. Furthermore, long-term CR elevates adropin gene levels in a tissue-specific manner through the transcriptional control of *ENHO* genes, suggesting that adropin gene mediates key metabolic adaptations to dietary restriction [101].

##### Adropin, a Metabolic Modulator

From a metabolic perspective, adropin improves insulin sensitivity by repressing hepatic gluconeogenesis and enhancing glucose uptake in peripheral tissues [100]. This involves sensitization of insulin signaling pathways, including Akt phosphorylation, as well as the upregulation of GLUT4-mediated glucose transport [101]. In addition to its role in glucose regulation, adropin supports lipid homeostasis by decreasing plasma triglycerides, total cholesterol, and low-density lipoprotein cholesterol levels, accompanied by an elevation in high-density lipoprotein cholesterol levels, leading to an overall improvement in plasma lipid composition [102].

Adropin also enhances cardiac energy metabolism, leading to improved myocardial efficiency, contractile performance, and coronary blood flow, highlighting its integrated role in coupling energy substrate regulation with cardiac function [100]. Concurrently, adropin mitigates vascular inflammation by downregulating tumor necrosis factor-alpha and interleukin-6; additionally, it augments eNOS activity to preserve endothelial function [177,178].

In summary, these reports indicate that adropin is a central microprotein that mediates the effects of CR, acting as a multifaceted metabolic regulator linked to lipid and glucose metabolism, thereby improving insulin sensitivity, cardiovascular protection, and inflammation control. Future studies leveraging these characteristics are recommended to validate the association between adropin-related genetic variants and metabolic diseases using human cohort studies and genome-wide association study linkage analyses. Furthermore, studies linking these findings with dietary intervention strategies, such as intermittent fasting or specific nutrient restrictions, that can replace the effects of CR are recommended.

### 6.4. Nutrient-Sensitive Peptides as Endocrine Regulators

#### 6.4.1. Nnat Links Glucose Sensing

*Nnat* is an imprinted gene, meaning that only one maternal or paternal line is activated, whereas the other is repressed by epigenetic mechanisms such as DNA methylation [103]. Imprinted genes significantly influence metabolic regulation and cellular growth. Among them, the *Nnat* gene encodes a β-cell-enriched microprotein that modulates insulin production and secretory function [74].

Importantly, *Nnat* gene deletion in pancreatic β cells was shown to disrupt glucose-stimulated insulin secretion in β-cell-specific knockout mice [74], which reported that these animals exhibited glucose intolerance in a nutrient-rich environment, despite normal food intake and body weight, which suggests that *Nnat* serves a critical role in β-cell physiology, rather than in systemic energy balance [74]. Furthermore, *Nnat* interacts with the signal peptidase complex, facilitating the cleavage of the proinsulin signal peptide and ensuring proper insulin maturation at the molecular level. Without *Nnat*, this process would have been less efficient, resulting in lower insulin levels and slower insulin secretion. Importantly, *Nnat* expression was upregulated by glucose, demonstrating its role as a regulator of nutrient sensitivity [74]. Beyond its function in pancreatic β cells, *Nnat* is also expressed in specific regions of the central nervous system, such as the hypothalamus, and its expression is modulated by leptin, implicating it in the regulation of feeding behavior and energy balance. These characteristics suggest a role for *Nnat* as an endocrine regulator of responses to nutritional signals.

#### 6.4.2. lncRNA TUNAR Encodes Dual Microproteins with Metabolic and Neural Functions

##### Microproteins as β-Cell and Neuronal Lineage Regulators

The lncRNA TUNAR, also referred to as *TUNA*, *HI-LNC78*, or *LINC00617*, was recently identified to encode a 48-amino acid microprotein termed *BNLN* [51]. *BNLN* localizes to the ER membrane of pancreatic β cells and contains a single-pass transmembrane domain. Functionally, *BNLN* overexpression reduces ER calcium levels, preserves ER homeostasis, and enhances glucose-stimulated insulin secretion in β cells [51]. These mechanisms have been validated experimentally in multiple animal models. Moreover, comparative analysis of pancreatic islets from mice fed a high-fat diet versus those fed a standard chow diet demonstrated that *BNLN* expression was downregulated in diet-induced obesity. Conversely, *BNLN* overexpression augments insulin secretion in islets from lean and obese mice, as well as in human samples [51]. Collectively, this study provided the first evidence that a lncRNA-encoded microprotein, *BNLN*, plays a critical role in maintaining pancreatic β-cell function, establishing a framework for understanding the physiological and pathological relevance of microproteins in diabetes.

##### Microprotein Complementing Metabolic Roles of pTUNAR

In addition to its metabolic role, this lncRNA encodes a second peptide, *pTUNAR*, which is abundant in neural tissues. pTUNAR resides in the ER and interacts with sarco/endoplasmic reticulum Ca^2+^-ATPase, thereby regulating intracellular calcium homeostasis [179]. Functional assays have revealed that pTUNAR overexpression reduces cytosolic calcium levels and suppresses neural differentiation and neurite outgrowth, whereas the loss of pTUNAR enhances neurogenesis both in vitro and in vivo [179]. These results suggest that pTUNAR acts as a fine tuner of calcium signaling pathways critical for neuronal lineage specification.

However, these findings provide proof-of-concept that microproteins derived from lncRNAs function as nutrient-sensitive endocrine regulators. BNLN responds to dietary and metabolic cues to optimize β-cell insulin secretion, directly linking ER calcium homeostasis to glucose metabolism [180]. In parallel, pTUNAR orchestrates neuronal development through calcium-dependent mechanisms, highlighting how a single lncRNA locus can generate multiple peptides with distinct, yet complementary, endocrine-like regulatory functions [87]. These studies highlight the potential of lncRNA-encoded microproteins as novel mediators of metabolic health and disease, with implications for diabetes, obesity, and neurodevelopmental disorders.

## 7. Challenges and Future Directions

Recent advances have enabled systematic identification of previously overlooked sORF-encoded peptides. Ribosome profiling (Ribo-seq) provides codon-level evidence of translation and remains the most sensitive platform for detecting microprotein-encoding sORFs [181]. Mass spectrometry-based proteomics, including targeted MS approaches, offers direct validation of microprotein expression at the peptide level [59]. Additional experimental tools—such as dual-luciferase reporter assays, endogenous epitope tagging, CRISPR-mediated knock-in strategies, and confocal imaging—facilitate validation of subcellular localization and biological function [105].

On the computational side, machine-learning and deep-learning algorithms, including LncCat, CPC, CPAT, PLEK, and ORF-attention-based predictors, integrate Ribo-seq enrichment, codon periodicity, and evolutionary conservation to identify novel microprotein candidates with high accuracy [182,183,184,185]. Together, these methods provide the combined proteogenomic framework necessary for discovering and validating functional microproteins.

### 7.1. Functional Validation Bottlenecks

Despite the identification of thousands of putative sORFs and microproteins across species, their functional validation remains challenging. Although recent ribosome profiling and mass spectrometry techniques provide evidence of translation, only a limited subset of these candidates has been experimentally validated [186].

This section highlights the unique features of microproteins that make them difficult to detect. First, considering traditional proteins, comprising hundreds to thousands of amino acids, facilitates antibody production, purification, and structural analyses. However, microproteins are short proteins, typically <100 amino acids, making them difficult to separate on protein gels and distinguish from other peptide fragments using mass spectrometry [187]. Second, microproteins lack homology to known proteins. Typically, protein function is predicted by comparing sequence similarity (homology) with known proteins. However, microproteins rarely resemble known proteins, making it difficult to infer their functions based solely on their sequences [59]. Third, the unfolded structure of microproteins presents a challenge. Generally, proteins have well-folded structures, allowing for structure-based function prediction. In contrast, microproteins often possess unfolded, disordered regions and function as short linear motifs that bind to specific proteins and regulate signal transduction [188]. Owing to these characteristics, microproteins often do not exhibit their functions independently, but rather interact with specific proteins in specific signaling pathways. Therefore, these functions are often overlooked in conventional validation experiments. For example, MOTS-c has many unfolded regions and does not maintain a stable tertiary structure [56]. It translocates into the cell nucleus and regulates the AMPK signaling pathway through interactions with specific transcription factors (binding via short linear motifs). In other words, it is a representative example of a protein that does not exhibit structural activity and requires binding to other proteins to exert its function [24]. Specifically, microproteins lack independent tertiary structures because of their disordered regions, making them difficult to detect using traditional structure-based function predictions and existing biochemical assays [189]. Furthermore, low expression levels and cell type specificity hinder reproducible detection [52]. These challenges create a gap between computational prediction and biological validation, highlighting the need for innovative experimental systems capable of capturing context-dependent functions of microproteins [52].

### 7.2. Lack of Annotation in Reference Genomes

A significant limitation of the current genome annotation is the systematic exclusion of sORFs. Contemporary genome annotation integrates multiple layers of evidence to catalog microprotein-encoding ORFs [182,190]. Ribosome profiling provides codon-resolved signatures of active translation, while mass spectrometry-based proteogenomics validates endogenous peptide expression [59,60,191]. Comparative genomic tools, such as PhyloCSF and RNAcode, identify conserved sORFs, and deep-learning predictors, including DeepRibo, together with curated repositories, such as SmProt, prioritize high-confidence candidates for functional characterization [37,39,61,192]. Accordingly, major reference genome projects (GENCODE, Ensembl, and RefSeq) now classify previously overlooked sORFs as uORFs, downstream ORFs, alternative ORFs, or novel coding sequence elements, reflecting a shift toward the systematic recognition of non-canonical coding sequences [182,190].

Historically, ORFs shorter than approximately 100 codons have been filtered out because of size limitations and the assumption of untranslatability, leaving many functional microproteins hidden within regions annotated as untranslated or untranslated [193]. Despite recent updates, reference genomes, such as GENCODE and RefSeq, fail to provide a comprehensive catalog of conserved sORFs across species, obscuring their evolutionary conservation and impeding their integration into the functional genomic and proteomic pipelines [194]. Emerging evidence suggests that 20–30% of the human transcriptome may harbor sORFs, but <1% are formally annotated [193]. Large-scale ribosome profiling and proteogenomic resources have highlighted hidden coding potential. Databases, such as sORFs.org (https://www.sorfs.org, accessed on 3 December 2025) and Open rot (https://www.openprot.org, accessed on 3 December 2025), catalog over 500,000 candidate ORFs, thousands of which have been confirmed to be translated, but most are not included in standard annotations [195,196]. To bridge this gap, an sORF or microprotein study requires experts in computational biology, molecular and cell biology, ribosome profiling, proteomics, genome annotation, structural biology, and medical research, indicating that a multidisciplinary collaboration is essential [197]. A multifaceted strategy is needed, including redesigning computational pipelines to integrate optimized ribosome profiling and comparative genomics signatures, building standardized large-scale ribosome profiling and proteogenomic databases, and iteratively updating reference genomes based on community-based open resources that follow FAIR (Findable, Accessible, Interoperable, Reusable) data principles [198,199].

### 7.3. Need for Proteogenomic and Ribosome-Profiling Integration

Although microproteins have emerged at the forefront of the “nutrient-translation” axis, much of the current evidence results from individual case studies such as SMIM26, NEMEP, and specific organization/condition-oriented results [22,23]. In order to quantitatively and systematically establish how these molecules link changes in nutritional availability to metabolic output directly from the ‘translation’ stage and mitochondrial sites, a proteogenomic approach that combines ribo-seq and precision proteomics is essential [62,200,201]. As outlined in this review, microproteins act at the ribosomal and mitochondrial levels through rapid, localized mechanisms, such as the SMIM26–SFXN1/2–mitoribosomal interaction and NEMEP–GLUT1/3 complex, functioning independently of slower transcriptional and signaling programs. These translationally mediated regulatory mechanisms operate on markedly different spatial and temporal scales than classical nutrient-responsive pathways such as AMPK- and PPAR-driven signaling [23,200]. To elucidate this difference at the full-length level, translation (ribosomal footsteps) and protein products (peptides/proteoforms) should be simultaneously observed in the same sample and along the same time axis.

## 8. Conclusions

Microproteins have emerged as direct translational nutrient sensors, thereby creating a paradigm shift in metabolic regulation. Unlike classical nutrient sensors, such as AMPK, PPAR, ChREBP, and HIF-1, which act through signaling cascades and transcriptional reprogramming, microproteins operate rapidly at the level of translation or within organelles [45,69]. Examples, such as SMIM26, which maintains mitochondrial translation under serine deprivation [45], and NEMEP, which enhances glucose uptake during embryonic differentiation [23], illustrate how microproteins directly couple nutrient availability with metabolic output. Other microproteins, including BRAWNIN and MTLN, integrate nutrient stress with mitochondrial respiration and fatty acid oxidation [30,31].

These findings suggest that microproteins are not only peripheral regulators but also essential components of nutrient sensing and energy homeostasis. They may confer therapeutic benefits in metabolic pathologies, such as obesity, diabetes, and hepatic steatosis, by modulating pathways that regulate glucose and lipid metabolism. Furthermore, their small size, rapid action, and tissue specificity make them attractive targets for precision nutrition and biomarker development [7,13,49].

Addressing functional validation barriers, improving genome annotations, and implementing proteogenomic integration are decisive steps. With these advances, microproteins can be fully recognized as fast-acting metabolic switches that complement classical pathways and provide new avenues for therapy and nutritional science.

## Figures and Tables

**Table 1:** Summary of microproteins discussed in this review, their nutrient-linked functions, and key references.

Microprotein	Genomic Origin	Primary Nutrient/ Stress Cue	Localization	Major Metabolic or Signaling Role	Physiological Context	Key References
SMIM26	Nuclear sORF [mitochondrial-targeted]	Serine deficiency; one-carbon metabolism	Mitochondria	Regulation of SFXN1/2; support of ND5 mitoribosomal translation; OXPHOS stabilization	Serine starvation, mitochondrial dysfunction	[22,45,72]
MTLN [Mitoregulin/MOXI]	LINC00116-encoded microprotein	FA oxidation state; mitochondrial lipid stress	Outer mitochondrial membrane	Modulation of FA β-oxidation; interaction with CPT1B/CYB5B; respiratory efficiency	Obesity; insulin resistance	[48,79,80]
Mitolamban [Mtlbn]	Nuclear-encoded microprotein [cardiac-enriched]	Mitochondrial respiratory stress	Inner mitochondrial membrane	Assembly/organization of Complex III and supercomplexes; redox balance	Cardiomyopathy; diabetes-related mitochondrial dysfunction	[81,82,83]
BRAWNIN	Nuclear sORF	Nutrient stress; AMPK activation	Inner mitochondrial membrane	Essential assembly factor for Complex III; supports OXPHOS	Mitochondrial dysfunction; metabolic stress	[49,84,85,86]
NEMEP	Nuclear-encoded microprotein	Growth-factor availability; glucose influx	Plasma membrane/endosomes	GLUT1/3 interaction; regulation of glucose uptake; glycolytic flux modulation	Embryogenesis; metabolic flexibility	[23,54]
MOTS-c	mtDNA-encoded [12S rRNA region]	Exercise; folate-cycle stress; glucose fluctuations	Cytosol → nucleus	AMPK activation; metabolic flexibility; stress adaptation	Obesity; type 2 diabetes	[24,56,87,88,89]
Humanin	mtDNA/NUMT	Oxidative stress	Cytosol, mitochondria	Anti-apoptotic and antioxidant signaling; mitochondrial protection	Aging; insulin resistance	[31,90,91,92,93,94]
Adropin	Nuclear-encoded secreted peptide [*ENHO*]	Feeding–fasting states; caloric restriction	Circulation	Substrate-use switching; lipid–glucose homeostasis	Dyslipidemia; NAFLD; insulin resistance	[95,96,97,98,99,100,101,102]
*Neuronatin* [*Nnat*]	Nuclear sORF [imprinted gene]	Glucose flux; ER Ca^2+^ handling	ER	Regulation of Ca^2+^-dependent insulin secretion; proinsulin processing	β-cell physiology; T2DM	[74,103]
BNLN	lncRNA-derived micropeptide	ER stress	ER	Ca^2+^ dynamics regulation; enhancement of GSIS	β-cell physiology	[51]
PIGBOS	Nuclear-encoded microprotein	ER–mitochondrial stress	Mitochondrial outer membrane [MOM]	Modulation of UPR; ER stress signaling	Metabolic stress	[58,104,105]
UFD1s	Nuclear-encoded [alternative splice-derived microprotein]	Metabolic stress; lipid overload	Cytosol	Modulates UFD1f/IPMK ubiquitination [K48/K63]; promotes autophagy and FA oxidation	NAFLD; NASH protection	[106]
MICT1	Nuclear-encoded	Cold exposure	Mitochondria	Enhancement of BAT thermogenesis; amplification of β-adrenergic signaling	Obesity; energy expenditure	[50,107]
FAM237B [Gm8773 peptide]	Nuclear-encoded secreted microprotein	Feeding state; adiposity signals	Hypothalamic arcuate nucleus; circulation	Central orexigenic signaling; regulation of food intake	Obesity; brain–adipose axis	[25,108]
ADM/ADM2	Nuclear-encoded peptide hormones	Circulating metabolic cues	Endocrine circulation	Regulation of insulin action; vascular perfusion; glucose delivery	Diabetes; metabolic syndrome	[109,110,111,112,113]

Abbreviations: FA, fatty acid; OXPHOS, oxidative phosphorylation; ER, endoplasmic reticulum; UPR, unfolded protein response; GSIS, glucose-stimulated insulin secretion; BAT, brown adipose tissue; NUMT, nuclear mitochondrial DNA segment; AMPK, AMP-activated protein kinase; MOM, mitochondrial outer membrane.

**Table 2:** Differences between conventional metabolic regulators and SMIM26.

Category	Conventional Metabolic Regulators	SMIM26
	(Kinases/Transcription Factors)	(Microprotein)
Target	Signal transduction/Transcriptional regulation	Direct regulation of ribosome/translation protein complexes
Level of Response	Transcription or post-translational signaling	Regulation at the translation level, directly influencing Complex I assembly
Nutrient Sensing	Typically mediated by AMPK, HIF-1-alpha, PPARs, etc.	Serine deficiency directly acts as a signal regulating SMIM26 expression
Mechanism	Indirect involves multiple transcriptional pathways	A novel regulatory axis via microprotein-mediated translational control

AMPK, AMP-activated protein kinase; PPAR, peroxisome proliferator-activated receptor; HIF-1, hypoxia-inducible factor-1.

**Table 3:** Temporal and mechanistic layers of nutrient-responsive regulation.

Level	Primary Sensor/Level	Typical Onset	Dominant Functional Role	Representative References
AMPK	Cellular energy charge; post-translational phosphorylation	Seconds–minutes	Acute metabolic triage: suppression of biosynthesis, stimulation of fatty acid oxidation, and enhanced glucose uptake	[18,133,136,137]
PPARs (α/γ/δ)	Lipid ligands; transcriptional regulation	Hours–days	Durable fuel selection and organ-level metabolic adaptation during fasting–feeding transitions	[19,78,135,138,139]
SMIM26	Nutrient-responsive peptides; translational control	Minutes	Direct modulation of mitochondrial translation (ND5) and respiratory chain activity	[22,45,72,143]
MOTS-c	Stress-induced mitochondrial peptides; peptide–kinase–transcription axis	Minutes–hours	Coupling of mitochondrial stress signals to AMPK and PPAR programs, thereby integrating acute and chronic metabolic regulation	[24,56,87,88]

AMPK, AMP-activated protein kinase; PPAR, peroxisome proliferator-activated receptor; MOTS-c, Mitochondrial Open Reading Frame of the 12S rRNA Type-c.

## Data Availability

No new data were created or analyzed in this study. Data sharing is not applicable to this article.

## Abbreviations

AICAR	5-Aminoimidazole-4-carboxamide ribonucleotide
ACC	Acetyl-CoA carboxylase
AC	Adenylate cyclase
ADM	Adrenomedullin
ADM2	Adrenomedullin 2
AMP	Adenosine monophosphate
AMPK	AMP-activated protein kinase
ATP	Adenosine triphosphate
bHLH	Basic helix–loop–helix
BAT	Brown adipose tissue
BNLN	β-cell and neuronal lineage regulator
CIII	Complex III (cytochrome bc1 complex)
CLR	Calcitonin receptor-like receptor
ChREBP	Carbohydrate response element-binding protein
CR	Caloric restriction
CPT1B	Carnitine palmitoyltransferase 1B
CPT1	Carnitine palmitoyltransferase 1
DUB	Deubiquitinating enzyme
eNOS	Endothelial nitric oxide synthase
ENHO	Energy homeostasis-associated gene
ER	Endoplasmic reticulum
FAO	Fatty acid oxidation
FAM210A	Family with sequence similarity 210 member A
Gαs	Guanine nucleotide-binding protein alpha subunit (stimulatory)
GLUT	Glucose transporter
GLUT1	Glucose transporter type 1
GLUT3	Glucose transporter type 3
GLUT4	Glucose transporter type 4
HIF-1	Hypoxia-inducible factor-1
HN	Humanin
ID	Inhibitor of DNA binding
JNK	c-Jun N-terminal kinase
KO	Knockout
lncRNA	Long non-coding RNA
MAPK	Mitogen-activated protein kinase
MAP3K20	Mitogen-activated protein kinase kinase kinase 20 (ZAKα)
MARCHF7	Membrane-associated ring-CH-type finger 7
mRNA	Messenger RNA
MIEF1	Mitochondrial elongation factor 1
MICT1	Microprotein for thermogenesis 1
MITR	Mitochondrial ribosome (mitoribosome)
MitoK_ATP	Mitochondrial ATP-sensitive potassium channel
MOTS-c	Mitochondrial open reading frame of the 12S rRNA type-c
mTORC1	Mechanistic target of rapamycin complex 1
Mtln	Mitoregulin
Mtlbn	Mitolamban
NADH	Nicotinamide adenine dinucleotide (reduced form)
NAFLD	Nonalcoholic fatty liver disease
NASH	Nonalcoholic steatohepatitis
ND5	NADH dehydrogenase subunit 5
NEMEP	Non-coding RNA expressed in mesoderm-inducing cells encoded with peptide
Nnat	Neuronatin
OXPHOS	Oxidative phosphorylation
pTUNAR	Peptide encoded by TUNAR lncRNA (neural isoform)
PKA	Protein kinase A
PP2B	Protein phosphatase 2B (Calcineurin)
PPAR	Peroxisome proliferator-activated receptor
PPARα	Peroxisome proliferator-activated receptor alpha
PPARγ	Peroxisome proliferator-activated receptor gamma
PPARδ	Peroxisome proliferator-activated receptor delta
PGC-1α	Peroxisome proliferator-activated receptor gamma coactivator 1-alpha
PTP1B	Protein tyrosine phosphatase 1B
RAMP	Receptor activity-modifying protein
RAMP2	Receptor activity-modifying protein 2
RAMP3	Receptor activity-modifying protein 3
RIIβ	Protein kinase A regulatory subunit II beta
RNA	Ribonucleic acid
RNP	Ribonucleoprotein
sORF	Small open reading frame
SERCA	Sarco/endoplasmic reticulum Ca^2+^-ATPase
SFXN	Sideroflexin
SFXN1	Sideroflexin 1
SFXN2	Sideroflexin 2
SMIM26	Small integral membrane protein 26
TCA	Tricarboxylic acid cycle
TG	Triglyceride
TUNAR	TUNA RNA (long non-coding RNA, also known as LINC00617)
T2DM	Type 2 diabetes mellitus
UCP1	Uncoupling protein 1
UFD1s	Ubiquitin fusion degradation 1 short isoform
UPR	Unfolded protein response
uORF	Upstream open reading frame
ZAKα	Sterile alpha motif and leucine zipper-containing kinase alpha

## References

[1] Hassel K.R., Brito-Estrada O., Makarewich C.A. (2023). Microproteins: Overlooked regulators of physiology and disease. iScience.

[2] Claverie J.M. (1997). Computational methods for the identification of genes in vertebrate genomic sequences. Hum. Mol. Genet..

[3] Mohsen J.J., Martel A.A., Slavoff S.A. (2023). Microproteins-Discovery, structure, and function. Proteomics.

[4] Li J., Qu L., Sang L., Wu X., Jiang A., Liu J., Lin A. (2022). Micropeptides translated from putative long non-coding RNAs. Acta Biochim. Biophys. Sin..

[5] Dong X., Zhang K., Xun C., Chu T., Liang S., Zeng Y., Liu Z. (2023). Small Open Reading Frame-Encoded Micro-Peptides: An Emerging Protein World. Int. J. Mol. Sci..

[6] Matsumoto A., Pasut A., Matsumoto M., Yamashita R., Fung J., Monteleone E., Saghatelian A., Nakayama K.I., Clohessy J.G., Pandolfi P.P. (2017). mTORC1 and muscle regeneration are regulated by the LINC00961-encoded SPAR polypeptide. Nature.

[7] Yan Y., Tang R., Li B., Cheng L., Ye S., Yang T., Han Y.C., Liu C., Dong Y., Qu L.H. (2021). The cardiac translational landscape reveals that micropeptides are new players involved in cardiomyocyte hypertrophy. Mol. Ther..

[8] Anderson D.M., Makarewich C.A., Anderson K.M., Shelton J.M., Bezprozvannaya S., Bassel-Duby R., Olson E.N. (2016). Widespread control of calcium signaling by a family of SERCA-inhibiting micropeptides. Sci. Signal..

[9] Wang B.Y., Gao Q., Sun Y., Qiu X.B. (2024). Biochemical targets of the micropeptides encoded by lncRNAs. Noncoding RNA Res..

[10] Lu Y., Ran Y., Li H., Wen J., Cui X., Zhang X., Guan X., Cheng M. (2023). Micropeptides: Origins, identification, and potential role in metabolism-related diseases. J. Zhejiang Univ. Sci. B.

[11] D’Lima N.G., Ma J., Winkler L., Chu Q., Loh K.H., Corpuz E.O., Budnik B.A., Lykke-Andersen J., Saghatelian A., Slavoff S.A. (2017). A human microprotein that interacts with the mRNA decapping complex. Nat. Chem. Biol..

[12] Kim S.J., Guerrero N., Wassef G., Xiao J., Mehta H.H., Cohen P., Yen K. (2016). The mitochondrial-derived peptide humanin activates the ERK1/2, AKT, and STAT3 signaling pathways and has age-dependent signaling differences in the hippocampus. Oncotarget.

[13] Yen K., Lee C., Mehta H., Cohen P. (2013). The emerging role of the mitochondrial-derived peptide humanin in stress resistance. J. Mol. Endocrinol..

[14] Makarewich C.A., Bezprozvannaya S., Gibson A.M., Bassel-Duby R., Olson E.N. (2020). Gene Therapy with the DWORF Micropeptide Attenuates Cardiomyopathy in Mice. Circ. Res..

[15] Staudt A.C., Wenkel S. (2011). Regulation of protein function by ‘microProteins’. EMBO Rep..

[16] Liu H., Wang S., Wang J., Guo X., Song Y., Fu K., Gao Z., Liu D., He W., Yang L.-L. (2025). Energy metabolism in health and diseases. Signal Transduct. Target. Ther..

[17] Chun Y., Kim J. (2021). AMPK-mTOR Signaling and Cellular Adaptations in Hypoxia. Int. J. Mol. Sci..

[18] Cui Y., Chen J., Zhang Z., Shi H., Sun W., Yi Q. (2023). The role of AMPK in macrophage metabolism, function and polarisation. J. Transl. Med..

[19] Barish G.D., Narkar V.A., Evans R.M. (2006). PPAR delta: A dagger in the heart of the metabolic syndrome. J. Clin. Investig..

[20] Heidenreich S., Weber P., Stephanowitz H., Petricek K.M., Schütte T., Oster M., Salo A.M., Knauer M., Goehring I., Yang N. (2020). The glucose-sensing transcription factor ChREBP is targeted by proline hydroxylation. J. Biol. Chem..

[21] Ziello J.E., Jovin I.S., Huang Y. (2007). Hypoxia-Inducible Factor (HIF)-1 regulatory pathway and its potential for therapeutic intervention in malignancy and ischemia. Yale J. Biol. Med..

[22] Nah J., Mahendran S., Kerouanton B., Cui L., Hock D.H., Cabrera-Orefice A., Dunlap K., Robinson D., Tung D.W.H., Leong S.H. (2025). Microprotein SMIM26 drives oxidative metabolism via serine-responsive mitochondrial translation. Mol. Cell.

[23] Ho C.W., Lee J.W., Shin C.H., Min K.W. (2025). LncRNA-Encoded Micropeptides: Expression Validation, Translational Mechanisms, and Roles in Cellular Metabolism. Int. J. Mol. Sci..

[24] Wan W., Zhang L., Lin Y., Rao X., Wang X., Hua F., Ying J. (2023). Mitochondria-derived peptide MOTS-c: Effects and mechanisms related to stress, metabolism and aging. J. Transl. Med..

[25] Martinez T.F., Lyons-Abbott S., Bookout A.L., De Souza E.V., Donaldson C., Vaughan J.M., Lau C., Abramov A., Baquero A.F., Baquero K. (2023). Profiling mouse brown and white adipocytes to identify metabolically relevant small ORFs and functional microproteins. Cell Metab..

[26] Saghatelian A., Couso J.P. (2015). Discovery and characterization of smORF-encoded bioactive polypeptides. Nat. Chem. Biol..

[27] van der Lee R., Buljan M., Lang B., Weatheritt R.J., Daughdrill G.W., Dunker A.K., Fuxreiter M., Gough J., Gsponer J., Jones D.T. (2014). Classification of Intrinsically Disordered Regions and Proteins. Chem. Rev..

[28] Jumper J., Evans R., Pritzel A., Green T., Figurnov M., Ronneberger O., Tunyasuvunakool K., Bates R., Žídek A., Potapenko A. (2021). Highly accurate protein structure prediction with AlphaFold. Nature.

[29] Mirdita M., Schütze K., Moriwaki Y., Heo L., Ovchinnikov S., Steinegger M. (2022). ColabFold: Making protein folding accessible to all. Nat. Methods.

[30] Kim J., DeBerardinis R.J. (2019). Mechanisms and Implications of Metabolic Heterogeneity in Cancer. Cell Metab..

[31] Hashimoto Y., Niikura T., Tajima H., Yasukawa T., Sudo H., Ito Y., Kita Y., Kawasumi M., Kouyama K., Doyu M. (2001). A rescue factor abolishing neuronal cell death by a wide spectrum of familial Alzheimer’s disease genes and Aβ. Proc. Natl. Acad. Sci. USA.

[32] Röhrig H., Schmidt J., Miklashevichs E., Schell J., John M. (2002). Soybean *ENOD40* encodes two peptides that bind to sucrose synthase. Proc. Natl. Acad. Sci. USA.

[33] Kondo T., Plaza S., Zanet J., Benrabah E., Valenti P., Hashimoto Y., Kobayashi S., Payre F., Kageyama Y. (2010). Small peptides switch the transcriptional activity of Shavenbaby during Drosophila embryogenesis. Science.

[34] Ingolia N.T., Hussmann J.A., Weissman J.S. (2019). Ribosome Profiling: Global Views of Translation. Cold Spring Harb. Perspect. Biol..

[35] Ma J., Ward C.C., Jungreis I., Slavoff S.A., Schwaid A.G., Neveu J., Budnik B.A., Kellis M., Saghatelian A. (2014). Discovery of human sORF-encoded polypeptides (SEPs) in cell lines and tissue. J. Proteome Res..

[36] Ingolia N.T., Brar G.A., Stern-Ginossar N., Harris M.S., Talhouarne G.J.S., Jackson S.E., Wills M.R., Weissman J.S. (2014). Ribosome Profiling Reveals Pervasive Translation Outside of Annotated Protein-Coding Genes. Cell Rep..

[37] Lin M.F., Jungreis I., Kellis M. (2011). PhyloCSF: A comparative genomics method to distinguish protein coding and non-coding regions. Bioinformatics.

[38] Olexiouk V., Crappé J., Verbruggen S., Verhegen K., Martens L., Menschaert G. (2015). sORFs.org: A repository of small ORFs identified by ribosome profiling. Nucleic Acids Res..

[39] Clauwaert J., Menschaert G., Waegeman W. (2019). DeepRibo: A neural network for precise gene annotation of prokaryotes by combining ribosome profiling signal and binding site patterns. Nucleic Acids Res..

[40] Hao Y., Zhang L., Niu Y., Cai T., Luo J., He S., Zhang B., Zhang D., Qin Y., Yang F. (2018). SmProt: A database of small proteins encoded by annotated coding and non-coding RNA loci. Brief. Bioinform..

[41] Almagro Armenteros J.J., Sønderby C.K., Sønderby S.K., Nielsen H., Winther O. (2017). DeepLoc: Prediction of protein subcellular localization using deep learning. Bioinformatics.

[42] Almagro Armenteros J.J., Tsirigos K.D., Sønderby C.K., Petersen T.N., Winther O., Brunak S., von Heijne G., Nielsen H. (2019). SignalP 5.0 improves signal peptide predictions using deep neural networks. Nat. Biotechnol..

[43] Szklarczyk D., Gable A.L., Nastou K.C., Lyon D., Kirsch R., Pyysalo S., Doncheva N.T., Legeay M., Fang T., Bork P. (2020). The STRING database in 2021: Customizable protein–protein networks, and functional characterization of user-uploaded gene/measurement sets. Nucleic Acids Res..

[44] Consortium T.G.O. (2020). The Gene Ontology resource: Enriching a GOld mine. Nucleic Acids Res..

[45] Konina D., Sparber P., Viakhireva I., Filatova A., Skoblov M. (2021). Investigation of LINC00493/SMIM26 Gene Suggests Its Dual Functioning at mRNA and Protein Level. Int. J. Mol. Sci..

[46] Roberts E.C., Deed R.W., Inoue T., Norton J.D., Sharrocks A.D. (2001). Id helix-loop-helix proteins antagonize pax transcription factor activity by inhibiting DNA binding. Mol. Cell. Biol..

[47] Norton J.D. (2000). ID helix-loop-helix proteins in cell growth, differentiation and tumorigenesis. J. Cell Sci..

[48] Zhang S., Guo Y., Fidelito G., Robinson D.R.L., Liang C., Lim R., Bichler Z., Guo R., Wu G., Xu H. (2023). LINC00116-encoded microprotein mitoregulin regulates fatty acid metabolism at the mitochondrial outer membrane. iScience.

[49] Zhang S., Reljić B., Liang C., Kerouanton B., Francisco J.C., Peh J.H., Mary C., Jagannathan N.S., Olexiouk V., Tang C. (2020). Mitochondrial peptide BRAWNIN is essential for vertebrate respiratory complex III assembly. Nat. Commun..

[50] Dinh J., Yi D., Lin F., Xue P., Holloway N.D., Xie Y., Ibe N.U., Nguyen H.P., Viscarra J.A., Wang Y. (2025). The microprotein C16orf74/MICT1 promotes thermogenesis in brown adipose tissue. EMBO J..

[51] Li M., Shao F., Qian Q., Yu W., Zhang Z., Chen B., Su D., Guo Y., Phan A.V., Song L.S. (2021). A putative long noncoding RNA-encoded micropeptide maintains cellular homeostasis in pancreatic β cells. Mol. Ther. Nucleic Acids.

[52] Makarewich C.A., Olson E.N. (2017). Mining for Micropeptides. Trends Cell Biol..

[53] Qian L., Zhu Y., Deng C., Liang Z., Chen J., Chen Y., Wang X., Liu Y., Tian Y., Yang Y. (2024). Peroxisome proliferator-activated receptor gamma coactivator-1 (PGC-1) family in physiological and pathophysiological process and diseases. Signal Transduct. Target. Ther..

[54] Fu H., Wang T., Kong X., Yan K., Yang Y., Cao J., Yuan Y., Wang N., Kee K., Lu Z.J. (2022). A Nodal enhanced micropeptide NEMEP regulates glucose uptake during mesendoderm differentiation of embryonic stem cells. Nat. Commun..

[55] Santoro M.M. (2025). The intersection between metabolism and translation through a subcellular lens. Trends Cell Biol..

[56] Lee C., Zeng J., Drew B.G., Sallam T., Martin-Montalvo A., Wan J., Kim S.J., Mehta H., Hevener A.L., de Cabo R. (2015). The mitochondrial-derived peptide MOTS-c promotes metabolic homeostasis and reduces obesity and insulin resistance. Cell Metab..

[57] Anderson D.M., Anderson K.M., Chang C.L., Makarewich C.A., Nelson B.R., McAnally J.R., Kasaragod P., Shelton J.M., Liou J., Bassel-Duby R. (2015). A micropeptide encoded by a putative long noncoding RNA regulates muscle performance. Cell.

[58] Chu Q., Martinez T.F., Novak S.W., Donaldson C.J., Tan D., Vaughan J.M., Chang T., Diedrich J.K., Andrade L., Kim A. (2019). Regulation of the ER stress response by a mitochondrial microprotein. Nat. Commun..

[59] Slavoff S.A., Mitchell A.J., Schwaid A.G., Cabili M.N., Ma J., Levin J.Z., Karger A.D., Budnik B.A., Rinn J.L., Saghatelian A. (2013). Peptidomic discovery of short open reading frame-encoded peptides in human cells. Nat. Chem. Biol..

[60] Ingolia N.T., Ghaemmaghami S., Newman J.R.S., Weissman J.S. (2009). Genome-Wide Analysis in Vivo of Translation with Nucleotide Resolution Using Ribosome Profiling. Science.

[61] Washietl S., Findeiss S., Müller S.A., Kalkhof S., von Bergen M., Hofacker I.L., Stadler P.F., Goldman N. (2011). RNAcode: Robust discrimination of coding and noncoding regions in comparative sequence data. RNA.

[62] Koch A., Gawron D., Steyaert S., Ndah E., Crappé J., De Keulenaer S., De Meester E., Ma M., Shen B., Gevaert K. (2014). A proteogenomics approach integrating proteomics and ribosome profiling increases the efficiency of protein identification and enables the discovery of alternative translation start sites. Proteomics.

[63] Steinberg G.R., Carling D. (2019). AMP-activated protein kinase: The current landscape for drug development. Nat. Rev. Drug Discov..

[64] Hardie D.G., Ross F.A., Hawley S.A. (2012). AMP-activated protein kinase: A target for drugs both ancient and modern. Chem. Biol..

[65] Issemann I., Green S. (1990). Activation of a member of the steroid hormone receptor superfamily by peroxisome proliferators. Nature.

[66] Uyeda K., Repa J.J. (2006). Carbohydrate response element binding protein, ChREBP, a transcription factor coupling hepatic glucose utilization and lipid synthesis. Cell Metab..

[67] Singh S., Sarkar T., Jakubison B., Gadomski S., Spradlin A., Gudmundsson K.O., Keller J.R. (2022). Inhibitor of DNA binding proteins revealed as orchestrators of steady state, stress and malignant hematopoiesis. Front. Immunol..

[68] Straub D., Wenkel S. (2017). Cross-Species Genome-Wide Identification of Evolutionary Conserved MicroProteins. Genome Biol. Evol..

[69] Yeasmin F., Yada T., Akimitsu N. (2018). Micropeptides Encoded in Transcripts Previously Identified as Long Noncoding RNAs: A New Chapter in Transcriptomics and Proteomics. Front. Genet..

[70] Ling F., Kang B., Sun X.H. (2014). Id proteins: Small molecules, mighty regulators. Curr. Top. Dev. Biol..

[71] Ovens A.J., Scott J.W., Langendorf C.G., Kemp B.E., Oakhill J.S., Smiles W.J. (2021). Post-Translational Modifications of the Energy Guardian AMP-Activated Protein Kinase. Int. J. Mol. Sci..

[72] Zhang T., Li Z., Li J., Peng Y. (2025). Small open reading frame-encoded microproteins in cancer: Identification, biological functions and clinical significance. Mol. Cancer.

[73] Wadding-Lee C.A., Makarewich C.A. (2025). Microproteins in Metabolism. Cells.

[74] Millership S.J., Da Silva Xavier G., Choudhury A.I., Bertazzo S., Chabosseau P., Pedroni S.M., Irvine E.E., Montoya A., Faull P., Taylor W.R. (2018). Neuronatin regulates pancreatic β cell insulin content and secretion. J. Clin. Investig..

[75] Xue K., Wu D., Wang Y., Zhao Y., Shen H., Yao J., Huang X., Li X., Zhou Z., Wang Z. (2022). The mitochondrial calcium uniporter engages UCP1 to form a thermoporter that promotes thermogenesis. Cell Metab..

[76] DeFronzo R.A. (2004). Pathogenesis of type 2 diabetes mellitus. Med. Clin. N. Am..

[77] Prentki M., Nolan C.J. (2006). Islet beta cell failure in type 2 diabetes. J. Clin. Investig..

[78] Kersten S. (2014). Integrated physiology and systems biology of PPARα. Mol. Metab..

[79] Krako Jakovljevic N., Pavlovic K., Jotic A., Lalic K., Stoiljkovic M., Lukic L., Milicic T., Macesic M., Stanarcic Gajovic J., Lalic N.M. (2021). Targeting Mitochondria in Diabetes. Int. J. Mol. Sci..

[80] Averina O.A., Permyakov O.A., Emelianova M.A., Guseva E.A., Grigoryeva O.O., Lovat M.L., Egorova A.E., Grinchenko A.V., Kumeiko V.V., Marey M.V. (2023). Kidney-Related Function of Mitochondrial Protein Mitoregulin. Int. J. Mol. Sci..

[81] Makarewich C.A., Munir A.Z., Bezprozvannaya S., Gibson A.M., Young Kim S., Martin-Sandoval M.S., Mathews T.P., Szweda L.I., Bassel-Duby R., Olson E.N. (2022). The cardiac-enriched microprotein mitolamban regulates mitochondrial respiratory complex assembly and function in mice. Proc. Natl. Acad. Sci. USA.

[82] Lowell B.B., Shulman G.I. (2005). Mitochondrial Dysfunction and Type 2 Diabetes. Science.

[83] Boudina S., Abel E.D. (2007). Diabetic Cardiomyopathy Revisited. Circulation.

[84] Wang Y., Shi Y., Li W., Han X., Lin X., Liu D., Lin Y., Shen L. (2024). Knockdown of BRAWNIN minimally affect mitochondrial complex III assembly in human cells. Biochim. Biophys. Acta Mol. Cell Res..

[85] Sergi D., Naumovski N., Heilbronn L.K., Abeywardena M., O’Callaghan N., Lionetti L., Luscombe-Marsh N. (2019). Mitochondrial (Dys)function and Insulin Resistance: From Pathophysiological Molecular Mechanisms to the Impact of Diet. Front. Physiol..

[86] Pacifici F., Malatesta G., Mammi C., Pastore D., Marzolla V., Ricordi C., Chiereghin F., Infante M., Donadel G., Curcio F. (2023). A Novel Mix of Polyphenols and Micronutrients Reduces Adipogenesis and Promotes White Adipose Tissue Browning via UCP1 Expression and AMPK Activation. Cells.

[87] Reynolds J.C., Lai R.W., Woodhead J.S.T., Joly J.H., Mitchell C.J., Cameron-Smith D., Lu R., Cohen P., Graham N.A., Benayoun B.A. (2021). MOTS-c is an exercise-induced mitochondrial-encoded regulator of age-dependent physical decline and muscle homeostasis. Nat. Commun..

[88] Kong B.S., Min S.H., Lee C., Cho Y.M. (2021). Mitochondrial-encoded MOTS-c prevents pancreatic islet destruction in autoimmune diabetes. Cell Rep..

[89] Gao Y., Wei X., Wei P., Lu H., Zhong L., Tan J., Liu H., Liu Z. (2023). MOTS-c Functionally Prevents Metabolic Disorders. Metabolites.

[90] Boutari C., Pappas P.D., Theodoridis T.D., Vavilis D. (2022). Humanin and diabetes mellitus: A review of in vitro and in vivo studies. World J. Diabetes.

[91] Kuliawat R., Klein L., Gong Z., Nicoletta-Gentile M., Nemkal A., Cui L., Bastie C., Su K., Huffman D., Surana M. (2013). Potent humanin analog increases glucose-stimulated insulin secretion through enhanced metabolism in the β cell. Faseb J..

[92] Muzumdar R.H., Huffman D.M., Atzmon G., Buettner C., Cobb L.J., Fishman S., Budagov T., Cui L., Einstein F.H., Poduval A. (2009). Humanin: A novel central regulator of peripheral insulin action. PLoS ONE.

[93] Lee H., Kang R., Bae S., Yoon Y. (2011). AICAR, an activator of AMPK, inhibits adipogenesis via the WNT/β-catenin pathway in 3T3-L1 adipocytes. Int. J. Mol. Med..

[94] Coradduzza D., Congiargiu A., Chen Z., Cruciani S., Zinellu A., Carru C., Medici S. (2023). Humanin and Its Pathophysiological Roles in Aging: A Systematic Review. Biology.

[95] Kumar K.G., Trevaskis J.L., Lam D.D., Sutton G.M., Koza R.A., Chouljenko V.N., Kousoulas K.G., Rogers P.M., Kesterson R.A., Thearle M. (2008). Identification of adropin as a secreted factor linking dietary macronutrient intake with energy homeostasis and lipid metabolism. Cell Metab..

[96] Hasanpour-Segherlou Z., Butler A.A., Candelario-Jalil E., Hoh B.L. (2024). Role of the Unique Secreted Peptide Adropin in Various Physiological and Disease States. Biomolecules.

[97] Stevens J.R., Kearney M.L., St-Onge M.P., Stanhope K.L., Havel P.J., Kanaley J.A., Thyfault J.P., Weiss E.P., Butler A.A. (2016). Inverse association between carbohydrate consumption and plasma adropin concentrations in humans. Obesity.

[98] Banerjee S., Ghoshal S., Stevens J.R., McCommis K.S., Gao S., Castro-Sepulveda M., Mizgier M.L., Girardet C., Kumar K.G., Galgani J.E. (2020). Hepatocyte expression of the micropeptide adropin regulates the liver fasting response and is enhanced by caloric restriction. J. Biol. Chem..

[99] Rooban S., Arul Senghor K.A., Vinodhini V.M., Kumar J.S. (2024). Adropin: A crucial regulator of cardiovascular health and metabolic balance. Metab. Open.

[100] Ali I., D’Souza C., Singh J., Adeghate E. (2022). Adropin’s Role in Energy Homeostasis and Metabolic Disorders. Int. J. Mol. Sci..

[101] Gao S., McMillan R.P., Zhu Q., Lopaschuk G.D., Hulver M.W., Butler A.A. (2015). Therapeutic effects of adropin on glucose tolerance and substrate utilization in diet-induced obese mice with insulin resistance. Mol. Metab..

[102] Ghoshal S., Stevens J.R., Billon C., Girardet C., Sitaula S., Leon A.S., Rao D.C., Skinner J.S., Rankinen T., Bouchard C. (2018). Adropin: An endocrine link between the biological clock and cholesterol homeostasis. Mol. Metab..

[103] Joseph R.M. (2014). Neuronatin gene: Imprinted and misfolded: Studies in Lafora disease, diabetes and cancer may implicate NNAT-aggregates as a common downstream participant in neuronal loss. Genomics.

[104] Qiu H., Schlegel V. (2018). Impact of nutrient overload on metabolic homeostasis. Nutr. Rev..

[105] Cheng H., Gang X., He G., Liu Y., Wang Y., Zhao X., Wang G. (2020). The Molecular Mechanisms Underlying Mitochondria-Associated Endoplasmic Reticulum Membrane-Induced Insulin Resistance. Front. Endocrinol..

[106] Li X., Wang X., Liu X., Shan G., Chen L. (2025). A UFD1 variant encoding a microprotein modulates UFD1f and IPMK ubiquitination to play pivotal roles in anti-stress responses. Nat. Commun..

[107] Pereira O.R., Serna J.D.C., Caldeira da Silva C.C., Camara H., Kodani S.D., Festuccia W.T., Tseng Y.H., Kowaltowski A.J. (2025). Mitochondrial ATP-sensitive K(+) channels (MitoK(ATP)) regulate brown adipocyte differentiation and metabolism. Am. J. Physiol. Cell Physiol..

[108] Narimatsu Y., Kato M., Iwakoshi-Ukena E., Moriwaki S., Ogasawara A., Furumitsu M., Ukena K. (2023). Neurosecretory Protein GM-Expressing Neurons Participate in Lipid Storage and Inflammation in Newly Developed Cre Driver Male Mice. Biomedicines.

[109] Cho H., Lai C.C., Bonnavion R., Alnouri M.W., Wang S., Roquid K.A., Kawase H., Campos D., Chen M., Weinstein L.S. (2025). Endothelial insulin resistance induced by adrenomedullin mediates obesity-associated diabetes. Science.

[110] Dai H.B., Wang F.Z., Kang Y., Sun J., Zhou H., Gao Q., Li Z.Z., Qian P., Zhu G.Q., Zhou Y.B. (2021). Adrenomedullin Attenuates Inflammation in White Adipose Tissue of Obese Rats Through Receptor-Mediated PKA Pathway. Obesity.

[111] Chauhan M., Ross G.R., Yallampalli U., Yallampalli C. (2007). Adrenomedullin-2, a Novel Calcitonin/Calcitonin-Gene-Related Peptide Family Peptide, Relaxes Rat Mesenteric Artery: Influence of Pregnancy. Endocrinology.

[112] Zhang S.Y., Xu M.J., Wang X. (2018). Adrenomedullin 2/intermedin: A putative drug candidate for treatment of cardiometabolic diseases. Br. J. Pharmacol..

[113] Lv Y., Zhang S.-Y., Liang X., Zhang H., Xu Z., Liu B., Xu M.-J., Jiang C., Shang J., Wang X. (2016). Adrenomedullin 2 Enhances Beiging in White Adipose Tissue Directly in an Adipocyte-autonomous Manner and Indirectly through Activation of M2 Macrophages. J. Biol. Chem..

[114] Weidemüller P., Kholmatov M., Petsalaki E., Zaugg J.B. (2021). Transcription factors: Bridge between cell signaling and gene regulation. Proteomics.

[115] Lee T.I., Young R.A. (2013). Transcriptional regulation and its misregulation in disease. Cell.

[116] Cui X., Wang J., Li K., Lv B., Hou B., Ding Z. (2024). Protein post-translational modifications in auxin signaling. J. Genet. Genom..

[117] Hardie D.G., Ross F.A., Hawley S.A. (2012). AMPK: A nutrient and energy sensor that maintains energy homeostasis. Nat. Rev. Mol. Cell Biol..

[118] Spriggs K.A., Bushell M., Willis A.E. (2010). Translational Regulation of Gene Expression during Conditions of Cell Stress. Mol. Cell.

[119] Dalla Venezia N., Vincent A., Marcel V., Catez F., Diaz J.J. (2019). Emerging Role of Eukaryote Ribosomes in Translational Control. Int. J. Mol. Sci..

[120] Greber B.J., Ban N. (2016). Structure and Function of the Mitochondrial Ribosome. Annu. Rev. Biochem..

[121] Poitevin F., Kushner A., Li X., Dao Duc K. (2020). Structural Heterogeneities of the Ribosome: New Frontiers and Opportunities for Cryo-EM. Molecules.

[122] Williams T.D., Rousseau A. (2024). Translation regulation in response to stress. Febs J..

[123] Uematsu S., Qian S.-B. (2025). Interpreting ribosome dynamics during mRNA translation. J. Biol. Chem..

[124] Darnell A.M., Subramaniam A.R., O’Shea E.K. (2018). Translational Control through Differential Ribosome Pausing during Amino Acid Limitation in Mammalian Cells. Mol. Cell.

[125] Snieckute G., Genzor A.V., Vind A.C., Ryder L., Stoneley M., Chamois S., Dreos R., Nordgaard C., Sass F., Blasius M. (2022). Ribosome stalling is a signal for metabolic regulation by the ribotoxic stress response. Cell Metab..

[126] Vind A.C., Genzor A.V., Bekker-Jensen S. (2020). Ribosomal stress-surveillance: Three pathways is a magic number. Nucleic Acids Res..

[127] Wu C.C.-C., Peterson A., Zinshteyn B., Regot S., Green R. (2020). Ribosome Collisions Trigger General Stress Responses to Regulate Cell Fate. Cell.

[128] Youn D., Kim B., Jeong D., Lee J.Y., Kim S., Sumberzul D., Ginting R.P., Lee M.-W., Song J.H., Park Y.S. (2025). Cross-talks between Metabolic and Translational Controls during Beige Adipocyte Differentiation. Nat. Commun..

[129] Rathore A., Chu Q., Tan D., Martinez T.F., Donaldson C.J., Diedrich J.K., Yates J.R., Saghatelian A. (2018). MIEF1 Microprotein Regulates Mitochondrial Translation. Biochemistry.

[130] Sareen Gropper J.S., Timothy C. (2008). Advanced Nutrition and Human Metabolism.

[131] Papakonstantinou E., Oikonomou C., Nychas G., Dimitriadis G.D. (2022). Effects of Diet, Lifestyle, Chrononutrition and Alternative Dietary Interventions on Postprandial Glycemia and Insulin Resistance. Nutrients.

[132] Wachsmuth H.R., Weninger S.N., Duca F.A. (2022). Role of the gut-brain axis in energy and glucose metabolism. Exp. Mol. Med..

[133] Garcia D., Shaw R.J. (2017). AMPK: Mechanisms of Cellular Energy Sensing and Restoration of Metabolic Balance. Mol. Cell.

[134] Saxton R.A., Sabatini D.M. (2017). mTOR Signaling in Growth, Metabolism, and Disease. Cell.

[135] Lehrke M., Lazar M.A. (2005). The many faces of PPARgamma. Cell.

[136] Lee M., Katerelos M., Gleich K., Galic S., Kemp B.E., Mount P.F., Power D.A. (2018). Phosphorylation of Acetyl-CoA Carboxylase by AMPK Reduces Renal Fibrosis and Is Essential for the Anti-Fibrotic Effect of Metformin. J. Am. Soc. Nephrol..

[137] Dagher Z., Ruderman N., Tornheim K., Ido Y. (2001). Acute Regulation of Fatty Acid Oxidation and AMP-Activated Protein Kinase in Human Umbilical Vein Endothelial Cells. Circ. Res..

[138] Ramakrishnan S.K., Khuder S.S., Al-Share Q.Y., Russo L., Abdallah S.L., Patel P.R., Heinrich G., Muturi H.T., Mopidevi B.R., Oyarce A.M. (2016). PPARα (Peroxisome Proliferator-activated Receptor α) Activation Reduces Hepatic CEACAM1 Protein Expression to Regulate Fatty Acid Oxidation during Fasting-refeeding Transition. J. Biol. Chem..

[139] Kersten S., Seydoux J., Peters J.M., Gonzalez F.J., Desvergne B., Wahli W. (1999). Peroxisome proliferator-activated receptor alpha mediates the adaptive response to fasting. J. Clin. Investig..

[140] Wang Y.X., Lee C.H., Tiep S., Yu R.T., Ham J., Kang H., Evans R.M. (2003). Peroxisome-proliferator-activated receptor delta activates fat metabolism to prevent obesity. Cell.

[141] Bougarne N., Weyers B., Desmet S.J., Deckers J., Ray D.W., Staels B., De Bosscher K. (2018). Molecular Actions of PPARα in Lipid Metabolism and Inflammation. Endocr. Rev..

[142] Ratman D., Mylka V., Bougarne N., Pawlak M., Caron S., Hennuyer N., Paumelle R., De Cauwer L., Thommis J., Rider M.H. (2016). Chromatin recruitment of activated AMPK drives fasting response genes co-controlled by GR and PPARα. Nucleic Acids Res..

[143] Kamradt M.L., Makarewich C.A. (2023). Mitochondrial microproteins: Critical regulators of protein import, energy production, stress response pathways, and programmed cell death. Am. J. Physiol. Cell Physiol..

[144] Hou M., Fan W., Zhong D., Dai X., Wang Q., Liu W., Li S. (2024). Ribosome Pausing Negatively Regulates Protein Translation in Maize Seedlings during Dark-to-Light Transitions. Int. J. Mol. Sci..

[145] Couso J.P., Patraquim P. (2017). Classification and function of small open reading frames. Nat. Rev. Mol. Cell Biol..

[146] Sivitz W.I., Yorek M.A. (2010). Mitochondrial dysfunction in diabetes: From molecular mechanisms to functional significance and therapeutic opportunities. Antioxid. Redox Signal..

[147] Sergiev P., Averina O., Golubeva J., Vyssokikh M., Dontsova O. (2025). Mitoregulin, a tiny protein at the crossroads of mitochondrial functioning, stress, and disease. Front. Cell Dev. Biol..

[148] Makarewich C.A. (2020). The hidden world of membrane microproteins. Exp. Cell Res..

[149] Huang N., Li F., Zhang M., Zhou H., Chen Z., Ma X., Yang L., Wu X., Zhong J., Xiao F. (2021). An Upstream Open Reading Frame in Phosphatase and Tensin Homolog Encodes a Circuit Breaker of Lactate Metabolism. Cell Metab..

[150] Puig M., Shubow S. (2025). Immunogenicity of therapeutic peptide products: Bridging the gaps regarding the role of product-related risk factors. Front. Immunol..

[151] Xiao W., Jiang W., Chen Z., Huang Y., Mao J., Zheng W., Hu Y., Shi J. (2025). Advance in peptide-based drug development: Delivery platforms, therapeutics and vaccines. Signal Transduct. Target. Ther..

[152] Govaere O., Hasoon M., Alexander L., Cockell S., Tiniakos D., Ekstedt M., Schattenberg J.M., Boursier J., Bugianesi E., Ratziu V. (2023). A proteo-transcriptomic map of non-alcoholic fatty liver disease signatures. Nat. Metab..

[153] Shi T.-t., Huang Y., Li Y., Dai X.-l., He Y.-h., Ding J.-c., Ran T., Shi Y., Yuan Q., Li W.-j. (2023). MAVI1, an endoplasmic reticulum–localized microprotein, suppresses antiviral innate immune response by targeting MAVS on mitochondrion. Sci. Adv..

[154] Chothani S., Ho L., Schafer S., Rackham O. (2023). Discovering microproteins: Making the most of ribosome profiling data. RNA Biol..

[155] Fan Y.H., Zhang S., Wang Y., Wang H., Li H., Bai L. (2024). Inter-organ metabolic interaction networks in non-alcoholic fatty liver disease. Front. Endocrinol..

[156] Pickart C.M. (2001). Mechanisms Underlying Ubiquitination. Annu. Rev. Biochem..

[157] Zhang L., Liu S., Zhao Q., Liu X., Zhang Q., Liu M., Zhao W. (2025). The role of ubiquitination and deubiquitination in the pathogenesis of non-alcoholic fatty liver disease. Front. Immunol..

[158] Zheng N., Shabek N. (2017). Ubiquitin Ligases: Structure, Function, and Regulation. Annu. Rev. Biochem..

[159] Komander D., Rape M. (2012). The Ubiquitin Code. Annu. Rev. Biochem..

[160] Zhang X., Xiao J., Jiang M., Phillips C.J.C., Shi B. (2025). Thermogenesis and Energy Metabolism in Brown Adipose Tissue in Animals Experiencing Cold Stress. Int. J. Mol. Sci..

[161] Osuna-Prieto F.J., Martinez-Tellez B., Sanchez-Delgado G., Aguilera C.M., Lozano-Sánchez J., Arráez-Román D., Segura-Carretero A., Ruiz J.R. (2019). Activation of Human Brown Adipose Tissue by Capsinoids, Catechins, Ephedrine, and Other Dietary Components: A Systematic Review. Adv. Nutr..

[162] Saito M., Matsushita M., Yoneshiro T., Okamatsu-Ogura Y. (2020). Brown Adipose Tissue, Diet-Induced Thermogenesis, and Thermogenic Food Ingredients: From Mice to Men. Front. Endocrinol..

[163] Qiu J., Khedr M.A., Pan M., Ferreira C.R., Chen J., Snyder M.M., Ajuwon K.M., Yue F., Kuang S. (2025). Ablation of FAM210A in Brown Adipocytes of Mice Exacerbates High-Fat Diet-Induced Metabolic Dysfunction. Diabetes.

[164] Zárate S.C., Traetta M.E., Codagnone M.G., Seilicovich A., Reinés A.G. (2019). Humanin, a Mitochondrial-Derived Peptide Released by Astrocytes, Prevents Synapse Loss in Hippocampal Neurons. Front. Aging Neurosci..

[165] Gong Z., Goetzman E., Muzumdar R.H. (2022). Cardio-protective role of Humanin in myocardial ischemia-reperfusion. Biochimica Biophys. Acta (BBA)—Gen. Subj..

[166] Cai H., Liu Y., Men H., Zheng Y. (2021). Protective Mechanism of Humanin Against Oxidative Stress in Aging-Related Cardiovascular Diseases. Front. Endocrinol..

[167] Guo J., Huang X., Dou L., Yan M., Shen T., Tang W., Li J. (2022). Aging and aging-related diseases: From molecular mechanisms to interventions and treatments. Signal Transduct. Target. Ther..

[168] Butler A.A., Zhang J., Price C.A., Stevens J.R., Graham J.L., Stanhope K.L., King S., Krauss R.M., Bremer A.A., Havel P.J. (2019). Low plasma adropin concentrations increase risks of weight gain and metabolic dysregulation in response to a high-sugar diet in male nonhuman primates. J. Biol. Chem..

[169] Miller B., Kim S.-J., Kumagai H., Mehta H.H., Xiang W., Liu J., Yen K., Cohen P. (2020). Peptides derived from small mitochondrial open reading frames: Genomic, biological, and therapeutic implications. Exp. Cell Res..

[170] Abulmeaty M.M.A., Almajwal A.M., Alnumair K.S., Razak S., Hasan M.M., Fawzy A., Farraj A.I., Abudawood M., Aljuraiban G.S. (2021). Effect of Long-Term Continuous Light Exposure and Western Diet on Adropin Expression, Lipid Metabolism, and Energy Homeostasis in Rats. Biology.

[171] Tripathi S., Maurya S., Singh A. (2025). Adropin mitigates reproductive and metabolic dysfunctions in streptozotocin induced hyperglycemic mice. J. Endocrinol..

[172] Mittal N., Ataman M., Tintignac L., Ham D.J., Jörin L., Schmidt A., Sinnreich M., Ruegg M.A., Zavolan M. (2024). Calorie restriction and rapamycin distinctly restore non-canonical ORF translation in the muscles of aging mice. Npj Regen. Med..

[173] Stevens J.R., Girardet C., Zhou M., Gamie F., Aggarwal G., McMillan R.P., Hulver M.W., Martinez L.O., van der Brug M., Vellas B. (2025). Adropin expression reflects circadian, lipoprotein, and mitochondrial processes in human tissues. Mol. Metab..

[174] Hofer S.J., Carmona-Gutierrez D., Mueller M.I., Madeo F. (2022). The ups and downs of caloric restriction and fasting: From molecular effects to clinical application. EMBO Mol. Med..

[175] Butler A.A., Havel P.J. (2025). Adropin: A cardio-metabolic hormone in the periphery, a neurohormone in the brain?. Peptides.

[176] Thapa D., Xie B., Manning J.R., Zhang M., Stoner M.W., Huckestein B.R., Edmunds L.R., Zhang X., Dedousis N.L., O’Doherty R.M. (2019). Adropin reduces blood glucose levels in mice by limiting hepatic glucose production. Physiol. Rep..

[177] Wang J., Ding N., Chen C., Gu S., Liu J., Wang Y., Lin L., Zheng Y., Li Y. (2025). Adropin: A key player in immune cell homeostasis and regulation of inflammation in several diseases. Front. Immunol..

[178] Zhang S., Chen Q., Lin X., Chen M., Liu Q. (2020). A Review of Adropin as the Medium of Dialogue between Energy Regulation and Immune Regulation. Oxidative Med. Cell. Longev..

[179] Senís E., Esgleas M., Najas S., Jiménez-Sábado V., Bertani C., Giménez-Alejandre M., Escriche A., Ruiz-Orera J., Hergueta-Redondo M., Jiménez M. (2021). TUNAR lncRNA Encodes a Microprotein that Regulates Neural Differentiation and Neurite Formation by Modulating Calcium Dynamics. Front. Cell Dev. Biol..

[180] Zhang T.N., Wang W., Yang N., Huang X.M., Liu C.F. (2020). Regulation of Glucose and Lipid Metabolism by Long Non-coding RNAs: Facts and Research Progress. Front. Endocrinol..

[181] Brar G.A., Weissman J.S. (2015). Ribosome profiling reveals the what, when, where, and how of protein synthesis. Nat. Rev. Mol. Cell Biol..

[182] Leong A.Z.-X., Lee P.Y., Mohtar M.A., Syafruddin S.E., Pung Y.-F., Low T.Y. (2022). Short open reading frames (sORFs) and microproteins: An update on their identification and validation measures. J. Biomed. Sci..

[183] Feng H., Wang S., Wang Y., Ni X., Yang Z., Hu X., Sen Y. (2023). LncCat: An ORF attention model to identify LncRNA based on ensemble learning strategy and fused sequence information. Comput. Struct. Biotechnol. J..

[184] Sun L., Liu H., Zhang L., Meng J. (2015). lncRScan-SVM: A Tool for Predicting Long Non-Coding RNAs Using Support Vector Machine. PLoS ONE.

[185] Wang L., Park H.J., Dasari S., Wang S., Kocher J.P., Li W. (2013). CPAT: Coding-Potential Assessment Tool using an alignment-free logistic regression model. Nucleic Acids Res..

[186] Pan J., Wang R., Shang F., Ma R., Rong Y., Zhang Y. (2022). Functional Micropeptides Encoded by Long Non-Coding RNAs: A Comprehensive Review. Front. Mol. Biosci..

[187] Andrews S.J., Rothnagel J.A. (2014). Emerging evidence for functional peptides encoded by short open reading frames. Nat. Rev. Genet..

[188] Trivedi R., Nagarajaram H.A. (2022). Intrinsically Disordered Proteins: An Overview. Int. J. Mol. Sci..

[189] Maiti S., Singh A., Maji T., Saibo N.V., De S. (2024). Experimental methods to study the structure and dynamics of intrinsically disordered regions in proteins. Curr. Res. Struct. Biol..

[190] Yandell M., Ence D. (2012). A beginner’s guide to eukaryotic genome annotation. Nat. Rev. Genet..

[191] Prensner J.R., Abelin J.G., Kok L.W., Clauser K.R., Mudge J.M., Ruiz-Orera J., Bassani-Sternberg M., Moritz R.L., Deutsch E.W., van Heesch S. (2023). What Can Ribo-Seq, Immunopeptidomics, and Proteomics Tell Us About the Noncanonical Proteome?. Mol. Cell. Proteom..

[192] Li Y., Zhou H., Chen X., Zheng Y., Kang Q., Hao D., Zhang L., Song T., Luo H., Hao Y. (2021). SmProt: A Reliable Repository with Comprehensive Annotation of Small Proteins Identified from Ribosome Profiling. Genom. Proteom. Bioinform..

[193] Valdivia-Francia F., Sendoel A. (2024). No country for old methods: New tools for studying microproteins. iScience.

[194] Pruitt K.D., Brown G.R., Hiatt S.M., Thibaud-Nissen F., Astashyn A., Ermolaeva O., Farrell C.M., Hart J., Landrum M.J., McGarvey K.M. (2014). RefSeq: An update on mammalian reference sequences. Nucleic Acids Res..

[195] Olexiouk V., Van Criekinge W., Menschaert G. (2018). An update on sORFs.org: A repository of small ORFs identified by ribosome profiling. Nucleic Acids Res..

[196] Brunet M.A., Brunelle M., Lucier J.-F., Delcourt V., Levesque M., Grenier F., Samandi S., Leblanc S., Aguilar J.-D., Dufour P. (2018). OpenProt: A more comprehensive guide to explore eukaryotic coding potential and proteomes. Nucleic Acids Res..

[197] Yang M., Xie Y., Wang L., Jungreis I., Ou T., Kellis M., Wang J., Zhu Y. (2025). Proteogenomics-enabled discovery of novel small open reading frame (sORF)-encoded polypeptides in human and mouse tissues. Nucleic Acids Res..

[198] Wilkinson M.D., Dumontier M., Aalbersberg I.J., Appleton G., Axton M., Baak A., Blomberg N., Boiten J.-W., da Silva Santos L.B., Bourne P.E. (2016). The FAIR Guiding Principles for scientific data management and stewardship. Sci. Data.

[199] Mudge J.M., Harrow J. (2016). The state of play in higher eukaryote gene annotation. Nat. Rev. Genet..

[200] Ahmadian M., Suh J.M., Hah N., Liddle C., Atkins A.R., Downes M., Evans R.M. (2013). PPARγ signaling and metabolism: The good, the bad and the future. Nat Med..

[201] Vrbnjak K., Sewduth R.N. (2024). Multi-Omic Approaches in Cancer-Related Micropeptide Identification. Proteomes.

